# A novel transcription factor SIPA1: identification and verification in triple-negative breast cancer

**DOI:** 10.1038/s41388-023-02787-3

**Published:** 2023-07-27

**Authors:** Lijuan Guo, Wanjun Zhang, Xue Zhang, Jun Wang, Jiaqi Nie, Xiaomeng Jin, Ying Ma, Shi Wang, Xinhong Zhou, Yilei Zhang, Yan Xu, Yoshimasa Tanaka, Jingping Yuan, Xing-Hua Liao, Yiping Gong, Li Su

**Affiliations:** 1 Key Laboratory of Molecular Biophysics of Ministry of Education, College of Life Science and Technology, Huazhong University of Science and Technology, Wuhan, 430074 China; 2 Department of Breast Surgery, Renmin Hospital of Wuhan University, Wuhan University, Wuhan, 430060 China; 3 Institute of Biology and Medicine, College of Life Sciences and Health, Wuhan University of Science and Technology, Hubei, 430081 P. R. China; 4 The Institute of Molecular and Translational Medicine, Department of Biochemistry and Molecular Biology, School of Basic Medical Sciences, Xi’an Jiaotong University Health Science Center, Xi’an, 710061 China; 5 Department of Immunology and Cell Biology, Graduate School of Medicine, Kyoto University, Yoshida, Sakyo-ku, Kyoto, 606-8501 Japan; 6 Center for Medical Innovation, Nagasaki University, 1-7-1, Sakamoto, Nagasaki, 852-8588 Japan; 7 Department of Pathology, Renmin Hospital of Wuhan University, Wuhan, China

**Keywords:** Breast cancer, Extracellular matrix

## Abstract

Transcription factors (TFs) regulate the expression of genes responsible for cell growth, differentiation, and responses to environmental factors. In this study, we demonstrated that signal-induced proliferation-associated 1 (SIPA1), known as a Rap-GTPase-activating protein, bound DNA and served as a TF. Importin β1 was found to interact with SIPA1 upon fibronectin treatment. A TGAGTCAB motif was recognized and bound by DNA-binding region (DBR) of SIPA1, which was confirmed by electrophoretic mobility shift assay. SIPA1 regulated the transcription of multiple genes responsible for signal transduction, DNA synthesis, cell adhesion, cell migration, and so on. Transcription of fibronectin 1, which is crucial for cell junction and migration of triple-negative breast cancer (TNBC) cells, was regulated by SIPA1 in a DBR-dependent manner both in vivo and in vitro. Furthermore, single-cell transcriptome sequencing analysis of specimens from a metastatic TNBC patient revealed that SIPA1 was highly expressed in metastatic TNBC. Hence, this study demonstrated that SIPA1 served as a TF, promoting TNBC migration, invasion, and metastasis.

## Introduction

Transcriptionfactors (TFs) are regulatory proteins for the spatial and temporal transcription of particular genes from deoxyribonucleic acid into corresponding ribonucleic acid by recognizing and directly binding to certain specific DNA sequences in the promoter region [1, 2]. TFs have been reported to play key roles in various aspects of life activities including embryonic development, the creation and maintenance of cell type- and tissue-specific patterns of protein synthesis and responses to cellular signaling [3, 4]. Functionally, a wide variety of TFs are involved in many human diseases such as congenital malformations, a myriad of benign and malignant neoplasms [5, 6]. Many TFs have been reported to be involved in carcinogenesis, and approximately 20% of oncogenes are estimated to be TFs, whose constitutive expression is necessary to support cancer cell growth, survival, and cellular transformation [7–12]. The identification and verification as potential cancer associated TFs should also present such functional features and explain mechanisms for cancer progression.

Signal-induced proliferation-associated 1 (SIPA1), initially identified as a Rap GTPase-activating protein (Rap-GAP), was recently found to play a key role in the progression of various tumors [13–21]. A line of evidence suggested that SIPA1 may regulate the transcription of various genes, such as *ITGB1*, *MYH9*, *HIF2a, ABCB1*, and *CD44* [22–25]. Ma et al. demonstrated that a stretch of the amino acids (140-179 aa) of SIPA1 functions as a non-typical nuclear localization signal region guiding SIPA1 protein into the nuclei, up-regulating *ABCB1* expression in breast cancer cells [26]. In addition, other studies showed that SIPA1 enhanced the promoter activity of certain genes in vitro [22, 24, 25]. Moreover, we previously found that an amino acid stretch 540-1042 of SIPA1 formed a complex with the promoter of *EPAS1*on the standard electrophoretic mobility shift assay (EMSA), indicating that SIPA1 may interact with DNA [24]. Accumulating evidence strongly suggests that SIPA1 could bind to DNA and regulate gene transcription. Based on these findings, we hypothesize that SIPA1 could serve as an unreported TF, which is critical for transcriptional regulation of specific genes.

SIPA1 has been widely studied in triple negative breast cancer (TNBC). Park et al. identified 23 genes, including SIPA1, on chromosome 19, as factors responsible for breast cancer metastasis [27]. Lu et al. reported that the upregulation of SIPA1 could increase the risk of breast cancer cell metastasis in individuals treated with 5-Aza-CdR [28]. Wang et al. found that SIPA1 increased the expression of SMAD2 and SMAD3, sustaining the stemness of breast cancer cells [23]. Aggressive lung metastasis took place in mice transplanted with SIPA1-expressing breast cancer cells, whereas the invasiveness was significantly attenuated by SIPA1-knockdown [27]. Zhang et al. demonstrated that the activity of Rap1 was not altered when SIPA1 was knocked down in MDA-MB-231 breast cancer cells, whereas the cell mobility was significantly impaired, suggesting that SIPA1 regulated the mobility of breast cancer cells in a Rap1-independent pathway [22]. Moreover, SIPA1 was shown to alter glucose metabolism, leading to breast cancer progression [24], and to aggravate the malignancy of breast cancer by enhancing *MYH9* in extracellular vesicles [25]. It is, therefore, imperative to verify that SIPA1 serves as a TF and regulates cancer progression in TNBC, which would allow us to further understand the onset and progression of TNBC and to develop a novel clinical means to treat TNBC.

In this study, SIPA1 was confirmed to be an unreported TF and promote the progression of TNBC. It was demonstrated that SIPA1 could promote the target gene transcription by directly binding to the promoter with a DNA-binding motif of TGAGTCAB in a sequence-specific manner via its DNA binding region (DBR, Ser764-Ala864 aa) of SIPA1. Deletion of DBR in SIPA1 protein resulted in the arrest of TNBC migration, invasion, and recurrence. SIPA1 might be a new effective therapeutic target for the development of TNBC therapy.

## Results

### SIPA1 is transported into nuclei via interaction with importin β1 in the presence of fibronectin

**Fig. 1:**
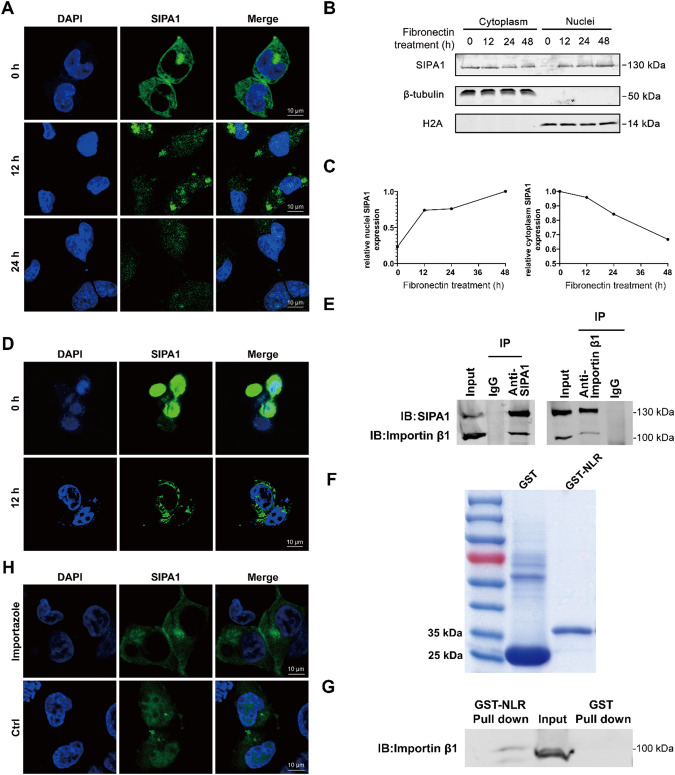
Fibronectin regulates SIPA1 trafficking between the cytoplasm and the nucleus. **A** Fluorescence microscopy imaging of SIPA1 in BT549 breast cancer cells which had been starved in FBS-free media and then treated with 5 μg/mL of fibronectin for 0, 12, and 24 h. SIPA1 is in green and nuclei in blue (stained with DAPI). Scale bar, 10 μm. **B**, **C** Cytoplasmic/nuclear localization of SIPA1 in BT549 cells that had been starved in FBS-free medium for 24 h and treated with 5 μg/mL of fibronectin. After incubation for 0, 12, 24, and 48 h, SIPA1 was detected by Western blot analysis. Nuclei: Nuclear fraction. Cytoplasm: Cytoplasmic fraction. β-tubulin was used as a representative cytoplasmic protein, and H2A as a nuclear protein (**B**). Quantification of SIPA1 protein expression levels (**C**). Nuclei SIPA1 expression (left), cytoplasm SIPA1 expression (right). **D** Fluorescence microscopy imaging of SIPA1 in BT549 cells that had been starved in FBS-free medium for 24 h, had been treated with 5 μg/mL fibronectin for 24 h and then change to FBS-free incubation in FBS-free medium for 0 and 12 h. SIPA1 is in green and the nuclei in blue (stained with DAPI). Scale bar, 10 μm. **E** Immunoprecipitation (IP) was performed using BT549 whole-cell lysates with anti-SIPA1 and anti-importin β1antibodies and Western blotting was conducted to detect the interaction between SIPA1 and importin β1. **F** GST and GST-NLR fusion proteins were expressed in *E. coli*, purified on an affinity column, resolved on SDS-PAGE, and detected by Coomassie-staining. Lane 1, GST; Lane2, GST-NLR, NLR: SIPA1(140-179 aa). **G** GST and GST-NLR fusion proteins were incubated with whole-cell lysates and Western blotting was performed to detect molecular interaction by using anti-importin β1 mAb. Endogenous importin β1 was used as a positive control. **H** Fluorescence microscopy imaging of SIPA1 in breast cancer cells incubated in fibronectin -containing media in the presence of 40 μg/mL of the importin β1 inhibitor importazole for 6 h. SIPA is in green and nuclei in blue (stained with DAPI). Scale bar, 10 μm.

Generally, TFs are located in the cytoplasm and translocated into the nuclei upon stimulation as non-constitutive nuclear proteins. SIPA1 was previously shown to localize in the nuclei of metastatic breast cancer cells in the presence of fibronectin or fetal bovine serum (FBS) [22]. To examine whether SIPA1 is a constitutive nuclear protein or is translocated into the nuclei upon stimulation, the localization of SIPA1 in BT549 cells was detected in the presence or absence of 5 μg/mL fibronectin through immunofluorescence imaging. As shown in Fig. 1A, SIPA1 was localized in the cytoplasm in the absence of serum. When BT549 cells were cultured in the presence of fibronectin for 12 h, SIPA1 protein was observed in both the nucleus and the cytoplasm. After 24 h incubation, a significant level of SIPA 1 was detected in the nucleus. SIPA1 expression in the nucleus and cytoplasm was further examined through Western blotting (Fig. 1B). After FBS starvation, most SIPA1 proteins were observed in the cytoplasmic fractions. When BT549 cells were incubated with fibronectin, SIPA1 was detected in the nuclear fractions and the expression levels were increased with time. Quantitative and spatio-temporal analyses of Western blot intensity data revealed that SIPA1 gradually decreased in the cytoplasmic fractions and increased progressively in the nuclear fractions over 48 h (Fig. 1C). Fibronectin also induced the translocation of SIPA1 into the nucleus in two other TNBC cell lines, MDA-MB-231 and SUM159 (Fig. S1). These results indicated that fibronectin elicited the translocation of SIPA1 from the cytoplasm into the nucleus, whereas SIPA1 remained in the cytoplasm in the absence of fibronectin. After 24 h fibronectin treatment, the culture medium was replaced by fibronectin-free medium, and the location of SIPA1 in BT549 cells was examined after additional 12 h incubation. As shown in Fig. 1D, most SIPA1 proteins were located in the cytoplasm. Thus, the SIPA1 protein is translocated into the nucleus in response to environmental stimuli, indicating that it is not a constitutive nuclear protein, like most TFs.

We then examined the mechanism by which SIPA1 was transported into the nucleus. In general, the nuclei-cytoplasmic transport is mediated by specific karyopherins, such as importins. Thus, a co-immunoprecipitation and proteomics assay were conducted to examine whether or not karyopherins are involved in the translocation of SIPA1 [22]. As shown in Table S1, importin β1 and importin 7 were found to bind with SIPA1. Cultured in the presence of fibronectin for 24 h, BT549 cells were harvested for Co-IP, and importin β1 was found co-immunoprecipitated with anti-SIPA1 antibody, suggesting that SIPA1 could interact with the nucleocytoplasmic transporter importin β1 (Fig. 1E). Conversely, SIPA1 was observed in the immunoprecipitated sample with anti-importin β1antibody (Fig. 1E). Ma et al. [26] reported that an N-terminal nuclear localization region (NLR, 140-179 aa) of the SIPA1 protein is crucial for its nuclear localization. We thus hypothesized that importin β1 might recognize a specific nuclear localization sequence (NLS) in the NLR of SIPA1, guiding SIPA1 translocation into the nucleus. To address this, GST-tagged SIPA1 NLR hinge (GST-NLR) was expressed and purified (Fig. 1F) and subjected to Co-IP. As shown in Fig. 1G, a band of importin β1 was observed for GST-NLR, but not for GST, indicating that importin β1 recognized and bound to the NLR of SIPA1. Next, to determine the role of importin β1 in the nuclear transport of SIPA1, 40 μg/mL importazole, an importin β1 inhibitor, was added to the fibronectin-containing culture medium and the SIPA1 localization in BT549 cells was monitored. As shown in Fig. 1H, the majority of SIPA1 was localized in the cytoplasm when importin β1 was inhibited. It is thus most likely that the NLR of SIPA1 protein interacted with the importin β1, which mediated the transportation of SIPA1 into the nucleus.

### SIPA1 directly binds the DNA containing a TGAGTCAB motif

**Fig. 2:**
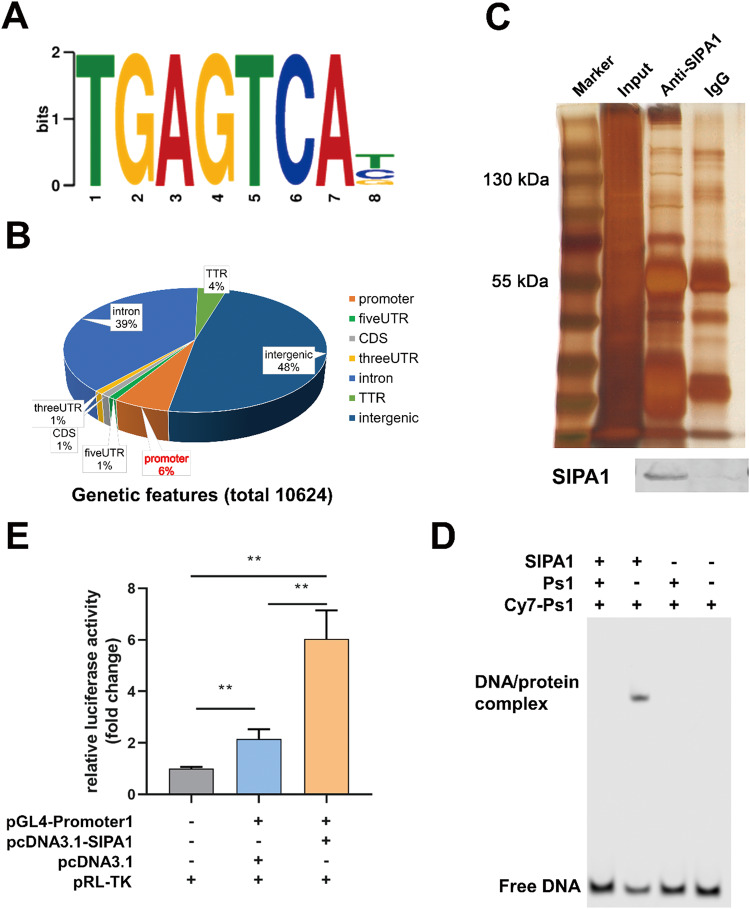
SIPA1 preferentially binds specific DNA motif. **A** Consensus motifs identified by MEME in ChIP-seq sequencing data in MDA-MB 231 cells. "B" means either T or C or G.The E-value is 4.9e-081. **B** Classification of SIPA1 binding sites in the human genome. The numbers of binding sites are indicated in the Dialogue bubble. **C** Protein G-immunoprecipitation assay was performed to enrich SIPA1 from BT549 lyzates and the co-precipitated proteins were resolved in SDS-polyacrylamide gel electrophoresis (SDS/PAGE) and silver stained, Lane1, input; Lane2, anti-SIPA1; Lane3, IgG. **D** DNA sequence bound with SIPA1 was evaluated using EMSA using endogenous SIPA1 proteins and cy7-labeled Ps1 containing a specific motif. **E** Dual Luciferase assay in HEK293T cells. HEK293T cells were co-transfected with SIPA1 expression plasmids and pGL4.10-Promoter1 luciferase reporter vector or pGL4.10 luciferase reporter vector (empty) were co-transfected into the cells. *P*-values were calculated using the unpaired two-tailed Student’s *t*-test (**p < 0.01).

We next examined whether SIPA1 recognizes and directly binds to specific sequences of DNA. Firstly, possible SIPA1-binding motifs were screened and mapped using the multiple expectation maximization for motif elicitation (MEME) algorithm on SIPA1-ChIP-seq data [23, 24]. A TGAGTCAB motif was identified as a consensus sequence site of SIPA1 (Fig. 2A). Among SIPA1-bound exonic and intronic sequences on DNAs, 633 reads were located in promoters, in which the binding sites of TFs existed, accounting for 6% of all sequences (Fig. 2B). The enriched sequences around the transcription start site (TSS) were identified in the SIAP1 ChIP-seq heatmap (Fig. S2A, S2B). Then, SIPA1 protein was expressed and enriched in vitro (Fig. 2C) for the standard EMSA. As shown in Fig. 2D, the promoter segment 1 (Ps1), a 126 bp DNA containing the TGAGTCAB motif randomly selected and cloned from the 633 promoter regions, was found to form a complex with SIPA1, whereas a shifted complex band failed to form in the absence of SIPA1. Several other fragments in the promoter region containing the TGAGTCAB motif also formed DNA-protein complexes with SIPA1 (Fig. S3A–S3D). These results suggested that SIPA1 recognized and bound the DNA segment Ps1.

Promotor 1 is the promotor region on specific genes containing Ps1. To further examine whether SIPA1, which might serve as a TF in the cells, regulates the transcriptional activity of promotor 1, promotor 1 with a size of 3000 bp was coloned from the upstream of *EPAS1* and dual-luciferase reporter gene assay was conducted using the promotor 1 sequence. In Fig. 2E, the transcription activity of the promoter 1 was significantly enhanced in the presence of SIPA1. Further, the promoter activities of *ITGB1*, *MYH9*, *ITGB4* and *TGFBI* gene were shown to be regulated by SIPA1 (Fig. S3E–S3H). Hence, it was confirmed that SIPA1 recognized and directly interacted with DNA.

We next searched the Eukaryotic Promotor Database (EPD) website and found that 4508 gene promoter regions contained the motif TGAGTCAB that might be recognized by SIPA1, suggesting that this motif frequently appeared in the promoter regions (Fig. S2C). Taken together, SIPA1 could function as a TF that directly binds to the DNA sequence containing the motif of TGAGTCAB and regulates the promoter activity.

### Ser764-Ala864 is indispensable for the interaction of SIPA1 with target gene promoters

**Fig. 3:**
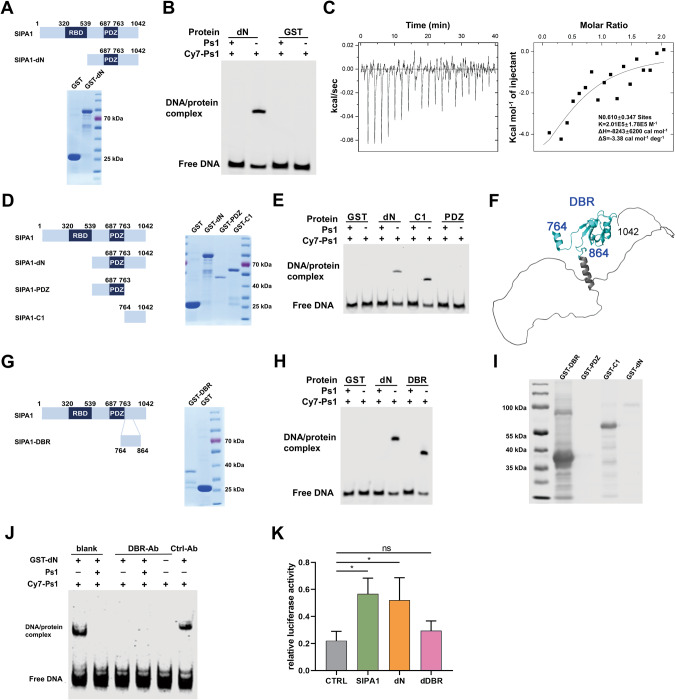
SIPA1 binds to DNA through the Ser764-Ala864 region. **A** Purification of dN fragments. Domain composition of full-length SIPA1 (top). Purification of dN fragments, Lane 1, elution fraction of GST protein; Lane2, elution fraction of GST- dN protein (bottom), dN: SIPA1(540-1042 aa). **B** EMSAs using Cy7- labeled Ps1, unlabeled Ps1 and purified GST-dN protein. **C** DNA binding affinity measurement of dN by ITC. The raw curves showed the change in thermal power with regard to time in the period of titration (left). The right curves showed the heat of reaction normalized with the molar ratio. Standard free energies of binding and entropic contributions were also obtained from the right curves. Thermodynamic parameters of the interaction between dN fragment and Ps1. All data were measured at 298 K in 25 mM HEPES, pH 7.5, 250 mM NaCl, 10 mM MgCl2, and 1 mM TCEP. **D** Purification of various SIPA1 fragments. Lane1, GST; Lane2, GST-dN; Lane3, GST-PDZ and Lane4, GST-C1. dN: SIPA1(540-1042 aa), PDZ: SIPA1(540-763 aa), and C1: SIPA1(764-1042 aa). **E** Comparison of Ps1 DNA binding with various SIPA1 fragments by EMSAs. 100 nM each of Cy7-labelled double stranded DNA was incubated with 200 nM of the dN, PDZ, or C1. **F** Structural representation of the SIPA1-C1 fragment. The DBR area is marked in green. **G** Purification of SIPA1 DBR fragment. Lane 1, GST-DBR; Lane2, GST. DBR: SIPA1(764-864 aa). **H** EMSAs by using Cy7-Ps1, unlabeled Ps1 and purified dN and DBR protein. **I** Western blot analysis of various purified SIPA1 fragments by anti-human DBR Ab. **J** EMSAs by using Cy7- labeled Ps1, unlabeled Ps1, GST-dN protein, with/without treatment of mouse anti-human DBR Abs, or control Ab. **K** Dual Luciferase assay in HEK293T cells. Plasmids of full-length SIPA1, DBR-del-SIPA1 (dDBR), N-del-SIPA1 (dN) and pGL4.10-Promoter1 luciferase reporter vector were co-transfected into HEK293T cells. Data were represented as means ± SD from triplicate samples, and three independent experiments. *P*-values were calculated using the unpaired two-tailed Student’s *t*-test (ns: not significant; *p < 0.05).

To identify the binding domain of SIPA1 protein to gene promoters, whether classical DNA-binding domains exist in SIPA1 was examined by blasting, while none was matched. Then, the protein structures of TFs that could bind DNA motif TGAGTCAB were examined. TFs with such a motif turned out to be closely related to the AP1 family, FOS-related proteins, and B-ATF family. The protein structures of DNA binding domains on these TFs were searched for SIPA1, but no hit was obtained. Since the potential binding motif was not explicit, the binding region might be comprised of high-dimensional structures of SIPA1. Then, as the amino acids stretch 540-1042 of SIPA1 seems to bind the promoter of *EPAS1* [24], the GST-tagged C-terminal domain of SIPA1 (SIPA1-dN, 540-1042 aa) was expressed in *E. coli* (Fig. 3A) and subjected to EMSA to examine whether SIAP1-dN interacted with the Ps1. SIPA1 formed a band complexed with cy7 labeled Ps1 (cy7-Ps1), and the addition of unlabeled Ps1 interfered the binding between SIPA1-dN and cy7-Ps1 (Fig. 3B). It is, therefore, likely that SIPA1-dN directly interacted with the DNA segment Ps1. To detect the interaction between SIPA1-dN and Ps1, isothermal titration calorimetry (ITC) assay was performed. As shown in Fig. 3C, the binding constant Ka of SIPA1-dN to Ps1 was calculated as 2.01E5 ± 1.78E5 M^−1^. To narrow down the DNA binding region of SIPA1, we further divided the amino acid stretch into two segments based on the structure of SIPA1-DN [29], the SIPA1-PDZ (540-763 aa) and SIPA1-C1(764-1042 aa). The segments were expressed in *E. coli* and purified, respectively, as shown in Fig. 3D. Like SIPA1-dN, SIPA1-C1 retarded the migration of Ps1 (Fig. 3E), demonstrating that SIPA1-C1, not the PDZ domain, directly interacted with Ps1.

To search for the DNA binding region in the SPA1-C1 region, a tertiary structure model was constructed. Several α-helices and β-sheets were predicted in the 764-864 aa region, whereas the 865-1042 aa region did not seem to form explicit stable secondary structures (Fig. 3F). Hence, the 764-864 aa stretch might play an essential role in the interaction between SIPA1 and DNA. To validate this hypothesis, we expressed and purified the GST-tagged 764-864 aa (GST-DBR) peptide (Fig. 3G) and examined their binding with Ps1 by EMSA. According to Fig. 3H, GST-DBR could directly bind Ps1, indicating that the DNA-binding domain of SIPA1 was located in the SIPA1-DBR region. We next examined the specificity of the binding by utilizing the newly prepared anti-SIPA1-DBR Ab (Fig. 3I). As shown in Fig. 3J, the anti-SIPA1-DBR Ab inhibited the interaction between SIPA1-dN and Cy7-Ps1, whereas a super shift band was formed when SIPA1-dN was pre-incubated with control anti-SIPA1 mAb. Furthermore, SIPA1 failed to enhance the promoter activity of Ps1 when the DBR region was deleted (Fig. 3K). Thus, SIPA1 specifically recognized and directly bound to the DNA motif via the DBR region, which determined the promotor activities of SIPA1.

### SIPA1 suppresses *FN1* transcription and regulates TNBC progression in vitro

**Fig. 4:**
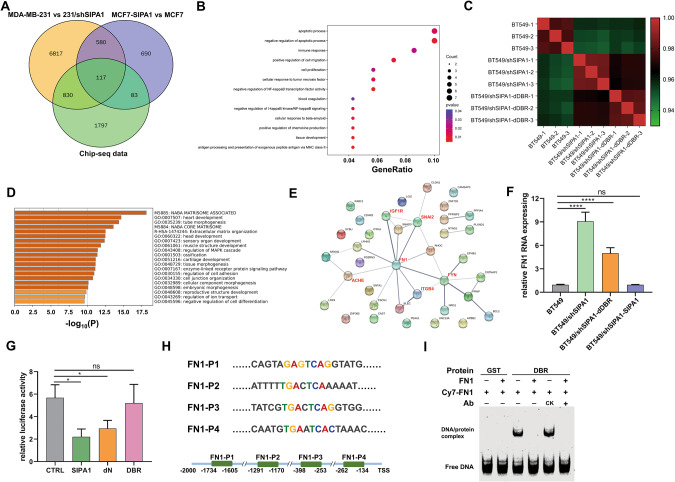
SIPA1 regulates FN1 transcription by binding to promoter region with the specific DNA motif. **A** Venn diagram illustrating the overlap of signature genes of ChIP-seq in MDA-MB-231 vs MDA-MB-231/shSIPA1 and in MCF7-SIPA1 vs MCF7. 117 signature genes shared by all the three groups. **B** Dot plot showing the enrichment analysis for Gene Ontology biological processes among group enrichment analyses of 117 genes. The size of dots represents the count of genes, and the spectrum of wogcolor indicates the mean *p*-values. **C** Sample correlation matrix: The Spearman correlation (R2) was calculated and visualized by color (green-red) in the matrix. Within the replicates for the individual sample groups, the correlation is higher than that between the sample groups. **D** GO biological process enrichment analyses of the 672 genes correlated with SIPA1 through the DBR domain. Top GO terms were listed with *p*-values. **E** Genes allocated to “cell junction organization” in D were plotted as a protein-protein interaction network. **F** mRNA detection of *FN1* in BT549, BT549/shSIPA1, BT549/shSIPA1-dDBR, and BT549/shSIPA1-SIPA1 cell lines. Data were shown as means ± s.d. The experiments were conducted in triplicate. α-Tubulin was included as an endogenous control. *P*-values were calculated using the unpaired two-tailed Student’s *t*-test (ns: not significant; ****p < 0.0001). **G** Dual Luciferase assay in HEK293T cells. SIPA1, dDBR-SIPA1, dN expression plasmids, and pGL4.10-FN1 luciferase reporter vector were co-transfected into the cells. Data were shown as means ± SD from triplicate samples, and three independent experiments. *P*-values were calculated using the unpaired two-tailed Student’s *t*-test (ns: not significant; *p < 0.05). **H** Oligonucleotide sequence used in the EMSA analysis (the core recognition element ‘GTACTCA’ is highlighted). FN1-P1, FN1-P2, FN1-P3, and FN1-P4 were DNA sequences from human *FN1* promoter. **I** EMSAs using Cy7-labeled FN1, unlabeled FN1 and purified DBR proteins.

As SIPA1 was widely studied in the development and metastasis of TNBC, the functional roles of SIPA1 as a TF were examined in TNBC. To address this, RNA-seq data of MDA-MB-231, MCF7, SIPA1-downregulated MDA-MB-231 (MDA-MB-231/shSIPA1), and SIPA1-overexpressing MCF7 (MCF7-SIPA1) breast cancer cell lines [24] together with ChIP-seq data were compared. We obtained a set of 117 genes that may be directly regulated by SIPA1. The GO analysis of this gene set indicated that SIPA1 might positively regulate cell migration (Fig. 4A, B). Furthermore, the *SIPA1* knocked-down BT549 cell line (BT549/shSIPA1) was established by introducing shRNA to BT549 cells. The rescued cell line was also established by over-expressing wild-type SIPA1 (BT549/shSIPA1-SIPA1). In addition, DBR region-deleted SIPA1-expressing BT549/shSIPA1 cells (BT549/shSIPA1-dDBR) were established (Fig. S4A, S4B). SIPA1 was found to be localized in the nucleus in BT549, BT549/shSIPA1-SIPA1, and BT549/shSIPA1-dDBR cells in the presence of fibronectin (Fig. S4C). The transcriptome sequencing was performed on BT549, BT549/shSIPA1, and BT549/shSIPA1-dDBR cells. BT549/shSIPA1 cells and BT549/shSIPA1-dDBR cells exhibited a high level of gene expression similarity, whereas BT549 and other two cell lines showed only a low level of similarity (Fig. 4C), indicating that DBR play an essential role in the regulation of target gene transcription by SIPA1. A total of 672 genes were differentially expressed between BT549 and BT549/shSIPA1 cells while similar between BT549/shSIPA1 and BT549/shSIPA1-dDBR cells (Fig. S5A). The GO analysis of these genes showed that they were closely related to “cell junction organization” and “extracellular matrix organization” (Fig. 4D). Also, the protein interaction network analysis of the genes revealed that FN1 was a pivotal factor on “cell junction organization” (Fig. 4E). TCGA-BRCA data indicated a negative correlation between SIPA1 and FN1 (Fig. S5B). To examine whether SIPA1 directly regulates the transcription of FN1, we analyzed the RNA expression levels of *SIPA1* in BT549, BT549/shSIPA1, BT549/shSIPA1-SIPA1, and BT549/shSIPA1-dDBR cells. As shown in Fig. 4F, the RNA transcription level of the *FN1* was significantly increased when *SIPA1* was knocked down. By contrast, when the SIPA1 protein expression level was rescued, the RNA transcription level of the *FN1* gene was significantly decreased. Besides, the RNA transcription level of the *FN1* gene was significantly higher in BT549/shSIPA1-dDBR cells than in BT549/shSIPA1-SIPA1 cells. These results strongly suggest that the expression of *FN1* was inhibited by wild-type SIPA1 in TNBC. We next examined whether the transcription of FN1 gene is directly regulated by SIPA1. On luciferase assay, SIPA1 significantly inhibited the transcriptional activity of *FN1*, which depended on DBR (Fig. 4G). The sequence alignment data revealed that four segments are similar to the SIPA1 binding motif in the *FN1* promoter (Fig. 4H). EMSA results indicated that the DBR region of SIPA1 protein directly interacted with the promoter of *FN1* (Fig. 4I). ChIP-PCR demonstrated that DBR-deleted SIPA1 failed to bind the *FN1* promoter in TNBC cells (Fig. S5C). Thus, it is most likely that SIPA1 protein directly binds to the promoter of *FN1* via the DBR region and inhibits the transcription of *FN1*. We next examined other genes involved in “cell junction organization” including *ITGB4, FYN, SNAI2, ACHE*, and *IGF1R*, for their expression patterns. *FYN*, *SNAI2*, and *ACHE* were co-expressed with SIPA1 in the TNBC cell line MDA-MB-231(Fig. S5D). Thus, SIPA1 could regulate the transcription of multiple target genes.

**Fig. 5:**
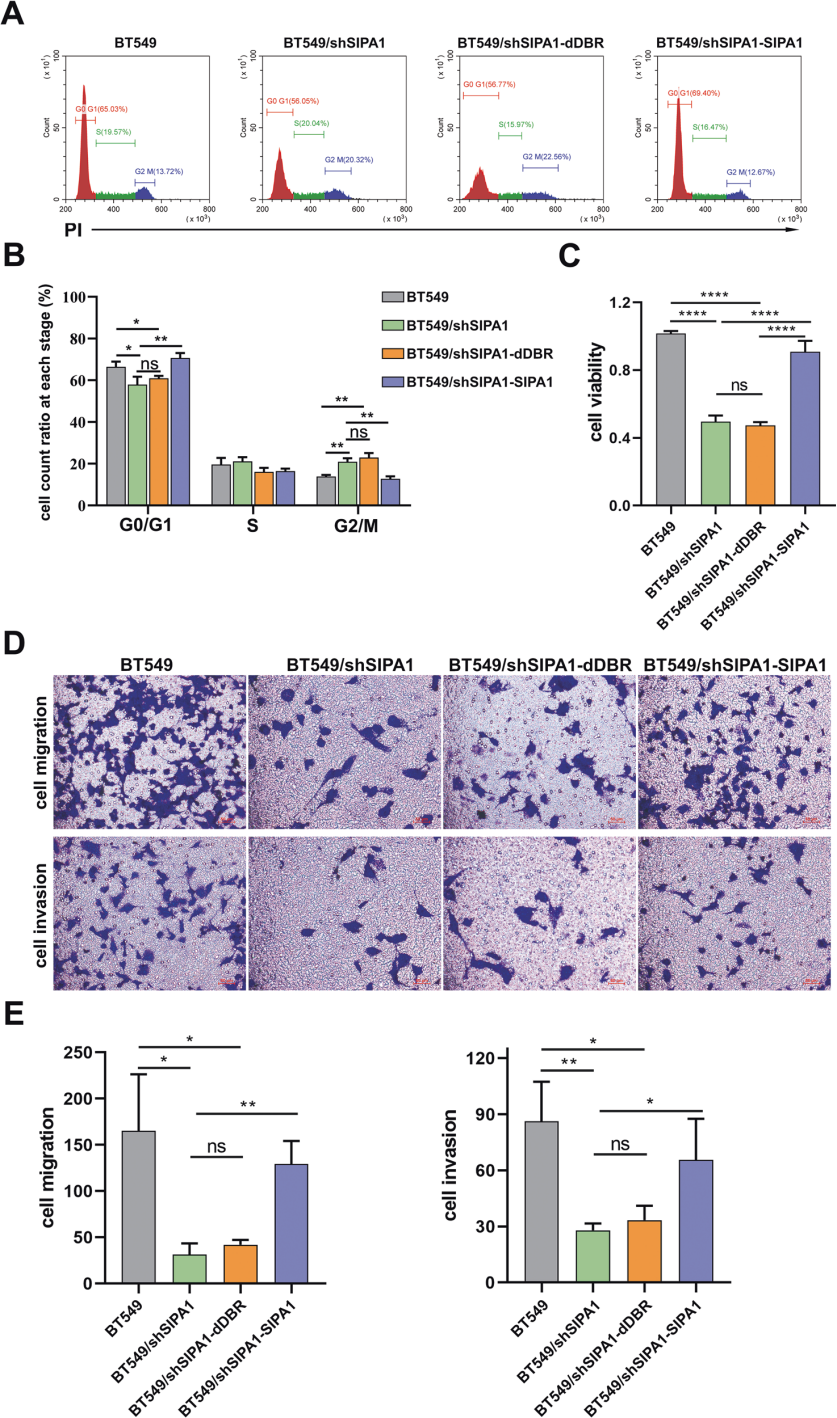
SIPA1 promotes breast cancer progression in vitro. **A**, **B** Flow cytometry was performed to examine the effects of SIPA1-DBR on cell cycle of BT549 (**A**) and Quantitative analyses of the cell cycle promotion and inhibition in BT549, BT549/shSIPA1, BT549/shSIPA1-dDBR and BT549/shSIPA1-SIPA1 cells (**B**). *P*-values were calculated using the unpaired two-tailed Student’s *t*-test (ns: not significant; *p < 0.05, **p < 0.01). **C** CCK8 assays for cell viability analysis. *P*-values were calculated using the unpaired two-tailed Student’s *t*-test (****p < 0.0001). **D**, **E** Transwell assay for migration detection and invasion analysis, Scale bar 30 µm (**D**) and Quantitative analyses of the migrated BT549, BT549/shSIPA1, BT549/shSIPA1-dDBR and BT549/shSIPA1-SIPA1 cells in transwell assay (**E**). *P*-values were calculated using the unpaired two-tailed Student’s *t*-test (ns: not significant; *p < 0.05; **p < 0.01).

We then examined whether the cell progression of TNBC is regulated by SIPA1 in a DBR domain-dependent manner. The proportion of BT549/shSIPA1 cells in the G0/G1 phase was decreased, whereas that in G2/M phase was increased (Fig. 5A, B). In addition, the proportion of SIPA1-overexpressing BT549/shSIPA1-SIPA1 cells in G2/M phase was decreased and that in G0/G1 phase was increased; but the BT549/shSIPA1-dDBR cells with over-expression of DBR-deleted SIPA1 showed no significant changes in the cell cycle status. Decreased cell viability was observed in both SIPA1-knocked-down (BT549/shSIPA1) and SIPA1-DBR-deleted BT549 cells, compared to that in wild-type SIPA1-expressing BT549 cells (BT549 or BT549/shSIPA1-SIPA1) (Fig. 5C). As shown in Fig. 5D, E, higher migration was observed in wild-type SIPA1-expressing BT549 cells than SIPA1-knocked down or SIPA1-DBR-deleted BT549 cells. The wound healing assay also showed similar results (Fig. S4D, S4E). Matrigel invasion assay was also used to examine the effects of SIPA1-DBR on invasive capacity of BT549 cells. The cell invasion was significantly lower in SIPA1-knocked down and SIPA1-DBR-deleted BT549 cells, when compared to wild-type SIPA1-expressing BT549 cells (Fig. 5D, E). These results suggested that SIPA1 could promote breast cancer cell migration and invasion in vitro, depending on its DNA-binding region.

### SIPA1 promotes TNBC progression in a DBR-dependent manner in vivo

**Fig. 6:**
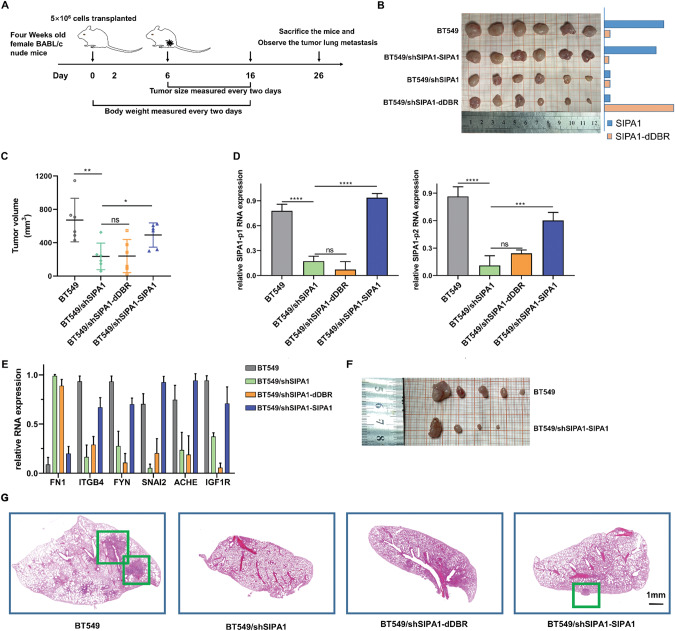
SIPA1 promotes TNBC progression in vivo. **A** Schematic figure showing experimental strategy and process. **B** Image of tumors collected from null mice treated with BT549, BT549/shSIPA1, BT549/shSIPA1-dDBR and BT549/shSIPA1-SIPA1 at day 16 after injection. The column represents the expression of SIPA1 or SIPA1-dDBR. **C** Quantification of tumor volume (length × width^2^/2 [mm^3^]) at various times after implantation. Data are mean ± SEM; *n* = 6. *P*-values were calculated using the unpaired two-tailed Student’s *t*-test (*p < 0.05, **p < 0.01). **D**, **E** Expression of SIPA1, SIPA1-dDBR (Left: the SIPA1-p1 was in the DBR region; right: the SIPA1-p2 was in the PDZ region) (**D**) and cell junction organization-related genes (**E**) determined by RT-qPCR in the tumors excised from mice. Data are mean ± SEM; *n* = 3. *P*-values were calculated using the unpaired two-tailed Student’s *t*-test (ns: not significant; ***p < 0.001, ****p < 0.0001). **F** Image of recurrent tumor collected from null mice treated with BT549 and BT549/shSIPA1-SIPA1 at the 10th day after surgical removal of tumor in situ. **G** Representative histopathological images of HE staining in the lungs were presented. Scale bar: 1 mm.

To explore TNBC progression in vivo, four kinds of cancer cell-derived xenograft (CDX) mouse models were established, in which mice were xenografted with (1) BT549 TNBC cells (*n* = 6), (2) BT549/shSIPA1 cells (*n* = 6), (3) BT549/shSIPA1-SIPA1 cells (*n* = 6), or (4) BT549/shSIPA1-dDBR cells (*n* = 6) (Fig. 6A) without significant body weight differences throughout the experiments (data not shown). For two groups inoculated with wild-type SIPA1-expressing cells (BT549 and BT549/shSIPA1-SIPA1 cells), the tumor growth rate was significantly higher, and the tumor size was much larger than that xenografted with BT549/shSIPA1 and BT549/shSIPA1-dDBR, and the growth rate of BT549/shSIPA1-dDBR cells was almost equivalent to that of BT549/shSIPA1 cells (Fig. 6B, C). These results suggested that SIPA1 promoted tumor progression in a DNA-binding region (DBR)-dependent manner in vivo.

Then, we examined mRNA expression levels of SIPA1 and cell junction organization-related genes in tumors. The mRNA expression of wild-type SIPA1 and DBR-deleted SIPA1 were detected (Fig. 6D). Upon measuring the expression of *FN1*, as well as *ITGB4, FYN, SNAI2, ACHE*, and *IGF1R*, decreased *FN1* and increased *ITGB4, FYN, SNAI2, ACHE*, and *IGF1R*, were observed in BT549 cells, and the opposite gene expression status was noted in BT549/shSIPA1 cells. The mRNA expression levels were restored by the over-expression of wild type SIPA1 in BT549/shSIPA1 cells, whereas the DBR-deleted SIPA1 failed to rescue the expression of these genes (Fig. 6E). Feeding the mice for another 10 days to observe tumor recurrence and pulmonary metastatic nodules at day 26, relapse was observed in BT549 group and BT549/shSIPA1-SIPA1 group (5 mice and 4 mice, respectively), whereas no recurrence was observed in BT549/shSIPA1 group and BT549/shSIPA1-dDBR group (Fig. 6F). While lung metastasis was not observed in BT549/shSIPA1-dDBR group, SIPA1-high expression groups exhibited tumor metastasis (Fig. 6G and S6). In zebrafish, cells expressing the wild-type SIPA1 protein also showed aggressive metastasis. Hence, SIPA1 with the DBR domain could promote metastasis and recurrence of TNBC in vivo.

### SIPA1 is over-expressed in metastatic TNBC patients by single cell RNA sequencing analysis

**Fig. 7:**
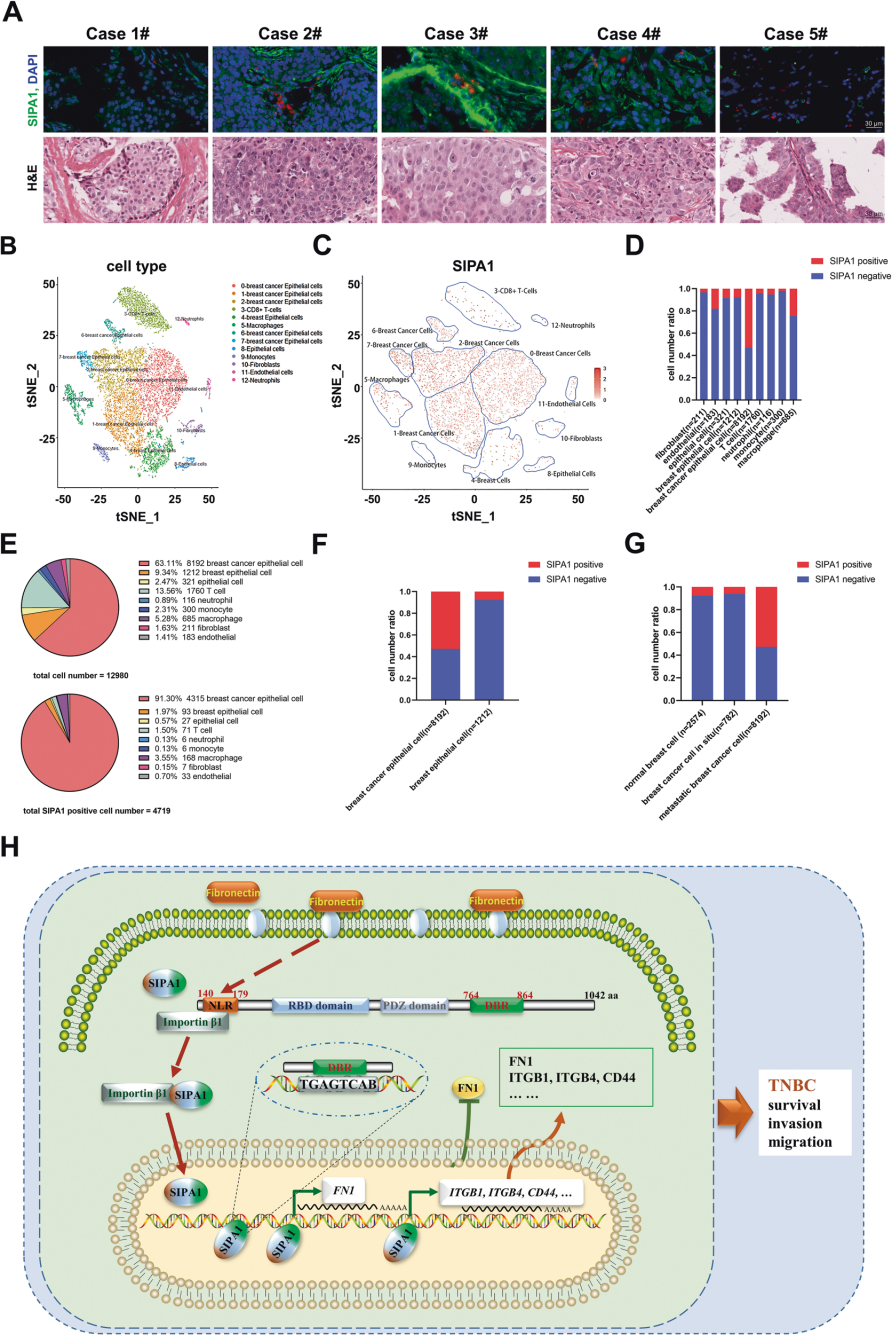
SIPA1 is highly expressed in tumor cells in TNBC patients. **A** The results of immunohistochemistry staining against SIPA1 in clinical breast cancer samples. SIPA1 protein is in green and nuclei in blue (stained with DAPI) (scale bars, 30 µm). **B** The tSNE of all 13098 cells that passed RNA QC and with > 500 genes detected from breast cancer patient. Cells are clustered into 13 groups according to known marker genes. **C** Expression levels of SIPA1 across 13098 single cells illustrated in t-SNE plots, color indicates mean expression levels within the cell type, the spectrum of color indicates the mean expression levels of the markers (log1p transformed). **D** Comparison of the partition of SIPA1-expressing cells with no expressing cells of each cluster. **E** Pie charts demonstrating distribution of the identified cell types across samples and the distribution of overexpression SIPA1 cell types. **F** Percentage of cells highly expressing SIPA1 in breast cancer cells versus that in breast epithelial cells. **G** Percentage of cells highly expressing SIPA1 in normal breast epithelial cells (GSM5022600) versus that in breast cancer cells in situ (GSM5374920) and metastasis. **H** Schematic illustration showing the roles of SIPA1 as a novel transcription factor by interacting with the cargo protein importin β1 and translocating into nucleus under fibronectin stimulation, and by directly binding to the TGAGTCAB motif containing-DNA via its DBR domain and enhancing the transcription activity of target genes. Dependent on its DBR domain, SIPA1 functions as a regulator by promoting cancer cell survival, invasion, migration, and recurrence of triple negative breast cancer.

We examined the expression and localization of SIPA1 in clinical samples by immunohistochemical staining and H&E staining to determine whether SIPA1 was located in the nuclei in the breast cancer. As shown in Fig. 7A, SIPA1 was detected in the nuclei in multiple breast cancer samples. To further confirm SIPA1 expression clinically, single-cell transcriptome sequencing assay was performed using a c samples taken during surgical removal of subcutaneous metastasis from a stage III TNBC patient with postoperative recurrence (Fig. 7B and S7A). SIPA1 was expressed in breast cancer cells, macrophages, endothelial cells, and T cells (Fig. 7C) and respective proportions were shown in Fig. 7D. The SIPA1 positive breast cancer cells account for more than 90% of SIPA1-positive cells, while breast cancer cells were just account for 63.11% of all cells (Fig. 7E). The proportion of SIPA1-positive cells in breast cancer cells ( > 50%) was higher than that in normal mammary epithelial cells ( < 10%), indicating that SIPA1 was aberrantly expressed in breast cancer cells, when compared to normal mammary epithelial cells (Fig. 7F). *FN1* expression of clusters with low SIPA1 expression was higher than that of clusters with high SIPA1 expression (Figs. S7B-S7E), which was consistent with the results in vitro. In addition, compared to orthotopic breast cancer samples and healthy breast samples from GEO datasets, the proportion of SIPA1-positive cells in metastatic TNBC cells was higher than that of those cells (Fig. 7G). The single-cell transcriptome sequencing assay revealed that SIPA1 was highly expressed in metastatic TNBC cells. Also confirming the genetic expression patterns of *FN1* related genes, *ITGB4*, *FYN, SNAI2, ACHE*, and *IGF1R* (Fig. 4E), the RNA transcription levels of these genes were closely related to that of SIPA1 (Fig. S8), so do the expression patterns of the reported genes (*ITGB1* [22], *EPAS1* [24]*, MYH9* [25], and *CD44* [23]) (Fig. S9). Thus, the clinical data are consistent with those of in vitro and in vivo results.

Taken together, SIPA1 was confirmed to serve as a TF, and the target genes of SIPA1 might be involved in the development and progression of TNBC.

## Discussion

Accumulating evidence indicates that SIPA1 is highly expressed in various solid tumors and can promote the expression of specific target genes. In the present study, SIPA1, a Rap GAP, was identified as a dual-function protein and serves as a transcription factor that promotes the progression of breast cancer (Fig. 7H). First, SIPA1 was localized in the cytoplasm under a serum-free condition but translocated into the nuclei in response to specific stimulatory signals. Next, it bound to a TGAGTCAB DNA motif of the promotor region of target genes through DBR (Ser764-Ala864) in SIPA1. Furthermore, *FN1* was identified as one of the target genes of SIPA1 by analysis through RNA-seq and EMSA. Finally, scRNA-seq of TNBC patient samples indicated a correlation between SIPA1 expression and breast cancer progression.

To date, several proteins have been reported to be dual-function proteins. β-Catenin regulates the co-ordination of cell-to-cell adhesion and functions as a TF [30]. Ceramide synthase Schlank is involved in the biosynthesis of ceramide as an enzyme and functions as a TF by binding promoter regions of lipases via its homeodomain [31]. The universal myo-inositol phosphate synthase is responsible for the biosynthesis of myo-inositol and has a function in transcriptional regulation of the histone methyltransferases in the nucleus [32]. Asr1, a plant TF, also functions as a chaperon-like protein [33, 34]. The lysyl-tRNA synthase is involved in the translation process and also participates in the transcriptional regulation of its target genes, such as microphathalmia TF and upstream stimulating factor 2 [35]. SIPA1 has been shown to be one of RapGAPs based on the existence of the Rap-binding domain. Besides the known function-associated domain, SIPA1 also have domains with unknown functions. In this study, we demonstrated that SIPA1 might have other functions based on its localization in the nuclei upon external stimuli.

Previous studies suggested a role of SIPA1 in the proliferation and metastasis of a variety of cancers. It was shown that SIPA1 overexpression could enhance breast cancer stemness, alter breast cancer metabolism, and enhance breast cancer drug resistance, thereby promoting breast cancer progression [23, 24, 26, 28]. Takahara et al. reported that SIPA1 promoted human oral squamous cell carcinoma via ITGB1 and MMP7 [36]. Shimizu et al. found that SIPA1 could regulate the interaction among extracellular matrix to promote human prostate cancer metastasis [20]. In non-small cell lung cancer (NSCLC), SIPA1 expression is correlated with that of receptor tyrosine kinases, especially HGF/MET and TJs, which regulates barrier function and cell invasion and overexpression of SIPA1 leads to the promotion of NSCLC metastasis [37]. This study confirmed that SIPA1 functioned as a TF to regulate cell junctional organization and extracellular matrix organization and to promote breast cancer progression. We propose a novel mechanism underlying SIPA1-mediated regulation of TNBC progression, in which the DBR domain on the SIPA1 protein directly interacts with the DNA motif in the promoter region of target genes. Based on these findings, it is most likely that SIPA1 might be a promising drug target for breast cancer and the present study provides a preclinical basis for the development of inhibitors of breast cancer cell metastasis.

## Materials and methods

### Cell culture and cell line derivation

Cell culture and the cell lines used were as described previously [24]. HEK293T and MDA-MB-231 were purchased from and certified by the China Center for Type Culture Collection (Wuhan, Hubei, China). BT549, a human breast cancer cell line, was procured from Procell (Wuhan, Hubei, China). HEK293T and BT549 cells were cultured in Dulbecco’s modified Eagle’s medium (DMEM, ThermoFisher Scientific, Waltham, MA) supplemented with 10% fetal bovine serum (FBS, Beyotime Biotechnology, Shanghai, China) and maintained at 37 °C with 5% CO_2_. MDA-MB-231 cells were cultured in RPMI-1640 (ThermoFisher Scientific) under the same culture conditions. To derivate BT549/shSIPA1 cell lines with stable expression of full-length or DBR-deleted SIPA1, BT549 cells were transformed with exogenous plasmids encoding full-length or truncated SIPA1. Finally, puromycin (0.4 μg/mL) was used to select relevant cell populations as described in the previous study [26].

### Xenograft tumor models

Animal experiments were conducted following the guidelines of Laboratory Animal Care formulated by the National Society of Medical Research and those for the US National Institutes of Health. The protocol was approved by the Animal Care and Use Committee of Huazhong University of Science and Technology (Ethic Code: S797). For xenograft studies, female BALB/c nude mice were divided into four groups at 5 weeks of age. The mice were randomized into control or treatment groups before cancer cell inoculation [24]. BT549, BT549/shSIPA1, BT549/shSIPA1-dDBR, and BT549/shSIPA1-SIPA1(5 × 10^6^/mouse) were subcutaneously injected with Matrigel (v/v 3:1) into the right mammary pad in each group. The tumor volume was determined every two days with a Vernier caliper according to the following formula: width^2^ × length/2 [mm^3^]). Finally, all the mice were sacrificed by cervical dislocation, and the tumors of each mouse were dissected, immersed in formalin.

### Breast cancer sample collection

The breast cancer specimens were collected from patients who had a mastectomy syndrome and completed an adjuvant chemotherapy in Renmin Hospital of Wuhan University, Wuhan, China. All subjects were informed, provided with written informed consent to participate in the study, and approved the use of the biopsy samples for pathological analyses, according to the Helsinki Declaration. The design of this study was ethically approved by the Clinical Research Ethics Committee of Hubei Cancer Hospital (ethical approval KYYW2018-023).

### Single-cell RNA sequencing and analysis

Tumors were bluntly resected, cut into small pieces, and digested using the Tumor Dissociation Kit in a gentle MACS Dissociator (Miltenyi, BergischGladbach, Germany) according to the manufacturer’s instruction. After digestion, all dead and dying cells were magnetically separated using a Dead Cell Removal Kit (Miltenyi, BergischGladbach, Germany). Single cells were partitioned using a Chromium Controller (10x Genomics), and gene expression sequencing libraries were generated using Chromium Single Cell 39 Library &Gel Bead Kit v3 (10x Genomics, Shanghai, China). The libraries were pooled and sequenced using an Illumina Nova Seq 6000 (Illumina). Raw base call files were demultiplexed with Cell Ranger 3.1.0 (10x Genomics) and bcl2fastq Conversion Software v2.20.0 (Illumina, San Diego, CA). The output results were analyzed using RStudio (Boston, MA). The data were analyzed using RStudio as follows. The outputs were filtered using a single-cell quality assurance/quality control filter to exclude doublets and multiples, and the cells with < 30 gene expressing per cell, and dead ones. The processed data were normalized by log normalization of gene expression and scaling to count per million and filtered out by feature (gene) not expressed in 99% of samples. Replicate samples were pooled and clustered with an unsupervised method based on principle component analysis with use of the top10 principal components. The clustered plots were visualized using t-distributed stochastic neighbor embedding (t-SNE) plot, in which cells were clustered with their shared nearest neighbor. Populations were determined by the expression of key markers, including KRT8, KRT18, KRT19, MUC1, and CD24 (epithelial cells). Chromosome copy number is used to distinguish breast cancer cells from normal epithelial cells.

### Plasmid constructs

For expression in *Escherichia coli* BL21, human cDNAs for SIPA1 variants were cloned into the pGEX4T-1 vector with an N-terminal glutathione S-transferase (GST) tag. The ΔN (dN, residues 540-1042 aa) constructs contained PDZ (Postsynaptic density 95; Discs large; Zonula occludens-1) and coiled-coil domains, in which the PDZ domain encompassed residues 540-764 aa and the coiled-coil (C1) domain consists of residues 764-1042 aa. The cDNA encoding ΔN, PDZ, C1 and DBR residue were cloned into pGEX4T-1 to express the respective GST-tagged proteins. The cDNA encoding DBR was cloned into pGEX4T-1 to express the His-tagged peptide. The cDNA for Sipa1, dDBR, was cloned into pcDNA3.1(+). The promoter DNA fragments for EPAS1 and FN1 were initially cloned into the pGL4.10 plasmid.

### Protein Expression and purification

Starting with a single transformed colony of *Escherichia coli* BL21 (DE3), expression cultures were grown in 2× YT rich media contain 50 µg/ml ampicillin at 37 °C to an OD_600_ of 0.8-1.0 and cold-shocked on ice/water bath for 20 min. Isopropyl--β-D-thiogalactopyranoside (IPTG) was added to a final concentration of 0.5 mM to induce protein expression. GST-C1, GST-DBR and His-DBR were expressed in *E. coli* BL21(DE3) at 37 °C for 3 h. GST-dN and GST-PDZ were expressed in *E. coli* BL21(DE3) at 16 °C for 48 h. The cells were collected by centrifugation and resuspended in buffer A (50 mM Na_2_HPO_4_, 50 mM KH_2_PO_4_, 500 mM NaCl, pH 7.4) containing complete ethylenediaminetetraacetic acid (EDTA)-free protease inhibitors. The cells were disrupted with a high-pressure crusher, and the lysate was clarified by centrifugation for 30 min at 20,000 g in a JA-25.5 rotor (Beckman coulter Inc., Brea CA) and applied to a Glutathione Sepharose 4 Fast Flow resin according to the manufacturer’s instructions (ThermoFisher Scientific). Next, the resin was washed with PBS and eluted with 10 mM reduced glutathione in phosphate-buffered saline (PBS). The final protein was concentrated to 4 mg/ml in a prewashed Amicon Ultra15 centrifugal filter (molecular weight cut off 10 kDa), flash-frozen in liquid nitrogen, and stored at −80 °C.

### Total RNA extraction, reverse transcription, and reverse transcription quantitative real-time PCR (RT-qPCR)

Total cell RNA was isolated using a TRIzol reagent (ThermoFisher Scientific) and subsequently transcribed to cDNA using a HiScript IIQ RT SuperMix for qPCR reagent Kit (Vazyme Biotech, Nanjing, China). RT-qPCR was performed using an SYBR Green II PCR kit (Vazyme Biotech, Nanjing) on an ABI7500 thermal cycler (Applied Biosystems, Waltham, MA). The primers were synthesized by TsingKe Biotech Company (Beijing, China). Differences in gene expression were calculated using the 2^-ΔΔCt^ method, in which the amplification of GAPDH was used for normalization.

### ChIP-seq

Chromatin Immuno Precipitation (ChIP) was performed according to the previously described procedure [31]. MDA-MB-231 cells (5 × 10^6^ cells) were individually cross-linked with 1% formaldehyde for 10 min at room temperature, and quenched with glycine at a final concentration of 0.125 M. Cross-linked cells were rinsed twice with cold PBS and then lysed in ChIP lysis buffer A (50 mM HEPES-KOH pH 7.5, 140 mM NaCl, 1 mM EDTA, pH 8.0, 10% glycerol, 0.5% NP-40, 0.25% Triton X-100, and protease inhibitor cocktail) for 10 min at 4 °C. Samples were centrifuged at 1400 × g for 5 min at 4 °C and the pellets were re-suspended and lysed in ChIP lysis buffer B (1% sodium dodecyl sulfate (SDS), 50 mM Tris-HCl pH 8.0, 10 mM EDTA and protease inhibitor cocktail) were lysed for 10 min at 4 °C. Mixed lysates were sonicated to yield 150–300 bp fragments using a Ultrasonic crusher (Scientz-IID) and centrifuged at 14,500 × g at 4 °C for 10 min. The supernatant was diluted by 10-fold with ChIP immunoprecipitation buffer (0.01% SDS, 1% Triton X-100, 2 mM EDTA, 50 mM Tris-HCl pH 8.0, 150 mM NaCl, and protease inhibitor cocktail). Lysates were incubated with the indicated antibodies overnight at 4 °C, and then protein G Sepharose (EMD, 3074030) were added to capture the immunoprecipitates. Beads were washed once with low salt buffer (0.1% SDS, 1% TritonX-100, 2 mM EDTA, 20 mM Tris–HCl, pH 8.0, and 150 mM NaCl), once with high salt buffer (0.1% SDS, 1% Triton X-100, 2 mM EDTA, 20 mM Tris-HCl pH 8.0,and 500 mM NaCl), once with LiCl buffer (0.25 M LiCl, 1% NP-40, 1% sodium deoxycholate, 1 mM EDTA and 10 mM Tris–HCl, pH 8.1), and twice with TE buffer (10 mM Tris-HCl and 1 mM EDTA pH, 8.0). Washed beads were eluted with fresh elution buffer (50 mM Tris-HCl, pH 8.0, 10 mM EDTA and 1.0% SDS) at 65 °C with vortex for 30 min. Supernatants were incubated at 65 °C for 8–16 h to reverse the crosslinking and release the immunoprecipitated DNA. After incubation with RNase A and proteinase K, DNA was purified with phenol: chloroform extraction and alcohol precipitation, and then sent for sequencing at Shanghai Biotechnology Corporation (Shanghai, China).

### Electrophoretic mobility shift assay (EMSA)

Eelectrophoretic mobility shift assay (EMSA) was performed as previously described [38]. In brief, the binding reactions were performed in a total mixture volume of 8 µL. The EMSA buffer used in this study contained 20 mM HEPES, pH 7.5, 200 mM NaCl, and 10 mM MgCl_2_. The labeled DNA was incubated with protein for 20 min on ice with a DNA concentration of 6 µM. The reaction mixture was loaded onto an 8% (w/v) native polyacrylamide gel using 0.5× TBE as the running buffer at 4 °C, 120 V, and 60 min. 5’-Cy7 labeled forward strand DNA oligos, and their unlabeled reverse complementary DNA strands were purchased from Tsingke (Wuhan, Hubei, China) and annealed. dsDNA (10 nM) was incubated with dN, C1, and DBR peptides. The bands were visualized using an Odyssey CLx (LI-COR), and band intensities were quantified with Image Studio^TM^ Lite software (LI-COR Biosciences, Nebraska, USA).

### Co-immunoprecipitation (Co-IP) assay

Approximately 10^7^ SIPA1-expressing cells were lysed in TNE lysis buffer (50 mM Tris-HCl pH 7.5, 150 mM NaCl, 0.5% NP40, 0.1% SDS, and 1 mM EDTA) containing protease inhibitor cocktail on ice for 15 min. Lysates were homogenized with a 0.4 mm needle and centrifuged at 13,000 × g for 15 min at 4 °C. Supernatants were either incubated with 10 μL of anti-SIPA1 antibody (ab189929, Abcam, Cambridge, UK) or covered with mouse IgG and rotated overnight at 4 °C. The following day, the mixed supernatants were incubated with 20 μL of protein G beads (3074030, Merck, Darmstadt, Germany) and rotated at 4 °C for 4 h. Then, they were washed five times with wash buffer (20 mM Tris-HCl, pH 7.4, 137 mM NaCl, 0.05% NP-40. and protease inhibitor cocktail), eluted by boiling in 50 μL SDS loading buffer, and followed by Western blot with antibodies (anti-SIPA1 mAb, ab189929, Abcam; anti-import β1 mAb, 8673, Cell Signaling Technology, Danvers, MA).

### Isothermal titration calorimetry assays

Isothermal titration calorimetry ITC (assays) were performed at 25 °C using a NANO ITC instrument (Waters Corp, Milford, MA). The purified protein was dissolved in an ITC buffer (20 mM HEPES, pH7.5, 250 mM NaCl, 10 mM MgCl_2_) at a concentration of 200 µM. The DNA duplex was dissolved in the same buffer to a concentration of 5 µM. The raw curves showed the change in thermal power to time in the period of titration (TOP). The bottom curves showed the heat of reaction normalized with the molar ratio. Standard free energies of binding and entropic contributions were also obtained from the bottom curves. Thermodynamic parameters of the interaction between DNA with SIPA1-DBR fragments were calculated based on the graphs.

### Luciferase assay

HEK293T cells were cultured in a complete high-glucose DMEM medium in 12-well plates. Transformation of HEK293T cells was performed using a Turbofect reagent (ThermoFisher Scientific), and the luciferase activity assays were conducted by a Flexstation3 multiplate reader (Molecular Devices, San Jose, CA). Briefly, HEK293T cells were co-transfected with 1.5 µg of reporter vector driven by the wild-type promoter, 1 µg of a vector coding SIPA1 or SIPA1-dDBR or a control vector, and 0.5 µg of empty vector pcDNA3.1(-). At 24 h post-transfection, the cells were harvested and disrupted with the lysis buffer provided by the manufacturer (ThermoFisher Scientific). After centrifugation at 1200 rpm for 5 min, 100 µL of the cell lysate was removed and used for luciferase assay through a dual-light detection system. Luciferase activity was normalized to that of galactosidase within the same sample. Relative luciferase activity was obtained by comparing the luciferase activity among wild-type SIPA1, SIPA1 knock-down (SIPA1-si), or SIPA1-dDBR expressing cells.

### Western blot (WB)

All samples were mixed with 5× loading buffer (10% SDS, 0.3 M Tris-HCl, pH 6.8, and 1.5 M dithiothreitol), boiled for 10 min, resolved on 10% slab gels for SDS-PAGE (NP0322BOX, ThermoFisher Scientific), and transferred onto polyvinylidene fluoride membranes (IPFL00010, Merck Millipore, Burlington, MA) through a Mini Trans Blot system (Bio-Rad Laboratories Inc., Hercules, CA). Then, the membranes were treated with TBS (10 mM Tris-HCl, pH 8.0, 150 mM NaCl, and 0.5% Tween-20) and 5% skimmed milk powder for 1 hour at room temperature. The membranes were then incubated with the indicated primary antibodies at 4 °C overnight, washed three times, incubated with a fluorescent dye-conjugated secondary antibody (926-32211, LI-COR Biosciences), and visualized using an Odyssey CLx (LI-COR Biosciences). The signal density was determined based on the corresponding band intensity of the scanned image.

### Microscopic fluorescence imaging

Immunofluorescence staining was performed as previously described. Cells expressing recombinant SIPA1 or SIPA1-DBD proteins were cultured on sterilized glass cover slides in 6-well culture plates and fixed with 4% (v/v) formaldehyde. To visualize SIPA1, the fixed cells were treated with 0.5% Triton X-100/PBS buffer and blocked with 3% BSA buffer for 2 h. Then, the cells were incubated with an anti-SIPA1 monoclonal antibody overnight. After being washed, the cells were treated with FITC-labeled secondary antibody (1:500, Proteintech, Rosemont, IL, USA). Fluorescence images were captured using an FV1000 Laser Scanning Confocal Microscope (Olympus Corp., Shinjuku, Tokyo, Japan) after staining the fixed cells with DAPI (1:1000) for 3 min at room temperature.

### Cell cycle test

Cells were fixed with 70% ethanol at 4 °C for 12 h, and subsequently reacted in staining buffer containing PI (7Sea Biotech, Shanghai, China) and RNase at 37 °C for 30 min. Cell cycle was tested by a Flow Cytometer (Beckman Coulter).

### Cell migration and invasion assay

Cell invasion was examined by transwell assays (Chamber with 8 µm membrane, ThermoFisher Scientific). Briefly, cells in serum-free medium were seeded into the upper chamber coated without or with Matrigel. The lower chamber contained 500 µL culture medium with 10% FBS. After incubation at 37 °C for 24 h, the migratory or invasive cells on the surface of the lower chamber were fixed with formaldehyde and stained with crystal violet, and finally photographed under an invert microscope.

### Transcriptome sequencing and analysis

Transcriptome sequencing and analysis were performed as previously described [24]. In brief, total RNA was isolated using TRIzol reagent (ThermoFisher Scientific). Transcriptome sequencing was performed by Novogene Co. Ltd. (Beijing, China). Gene expression levels for each transcript were estimated as the number of reads per kilobase of exon model per million mapped reads (RPKM); only uniquely mapped reads in exonic regions were used. A gene was considered differentially expressed if its expression differed between any two samples with a fold change > 2 and a *p*-value < 0.05. The DAVID (RRID: SCR_001881) online tool (https://david.abcc.ncifcrf.gov) was used for Gene Ontology (GO) enrichment analysis and KEGG (RRID: SCR_012773) pathway enrichment analysis. The STRING (RRID: SCR_005223) online tool (https://string-db.org) was used to evaluate the protein‒protein interactions of cluster genes.

### Statistical analyses

Statistical analyses were carried out using a Graphpad Prism (version 8.0, San Diego, CA) software. The two-tailed Student’s *t*-test was used for comparison between treatment and control groups. For multiple comparisons, the one-way ANOVA plus two-sided Tukey test was applied. Correlation analyses were performed using the Spearman correlation test. All values are expressed as the mean ± SD unless otherwise indicated, and *p* < 0.05 was considered significant. *p < 0.05; **p < 0.01; ***p < 0.001; ****p < 0.0001; ns, not significant (*p* > 0.05).

## Supplementary information

Supplementary material

## Data Availability

Single cell transcriptome sequencing datasets (GSE221731) and RNA-seq datasets (GSE221742) are available at GEO under the accession number GSE221743. The raw sequence data of MDA-MB-231, MDA-MB-231/shSIPA1, MCF7 and MCF7-SIPA1 in this paper (Yao et al., 2022) have been deposited in the Genome Sequence Archive (Chen et al., 2021) in National Genomics Data Center (CNCB-NGDC Members and Partners, 2021), China National Center for Bioinformation/Beijing Institute of Genomics, Chinese Academy of Sciences, under accession number HRA001265. The ChIP-seq data has been uploaded to GEO database (GSE124344). Other data presented in this study are available upon request from the corresponding author LS.
