# Supplementary material for: A novel transcription factor SIPA1: identification and verification in triple-negative breast cancer
Source: Oncogene. 2023 Jul 27;42(35):2641–54. doi: 10.1038/s41388-023-02787-3 (PMC10457189; doi:10.1038/s41388-023-02787-3)
Supplement: Supplementary file 1 — Supplementary material [file 41388_2023_2787_MOESM1_ESM.docx]

## Supplementary Material

**A Novel Transcription Factor SIPA1: Identification and Verification in Triple-Negative Breast Cancer**

Lijuan Guo, Wanjun Zhang, Xue Zhang, Jun Wang, Jiaqi Nie, Xiaomeng Jin, Ying Ma, Shi Wang, Xinhong Zhou, Yilei Zhang, Yan Xu, Yoshimasa Tanaka, Jingping Yuan, Xinghua Liao, Yiping Gong, Li Su


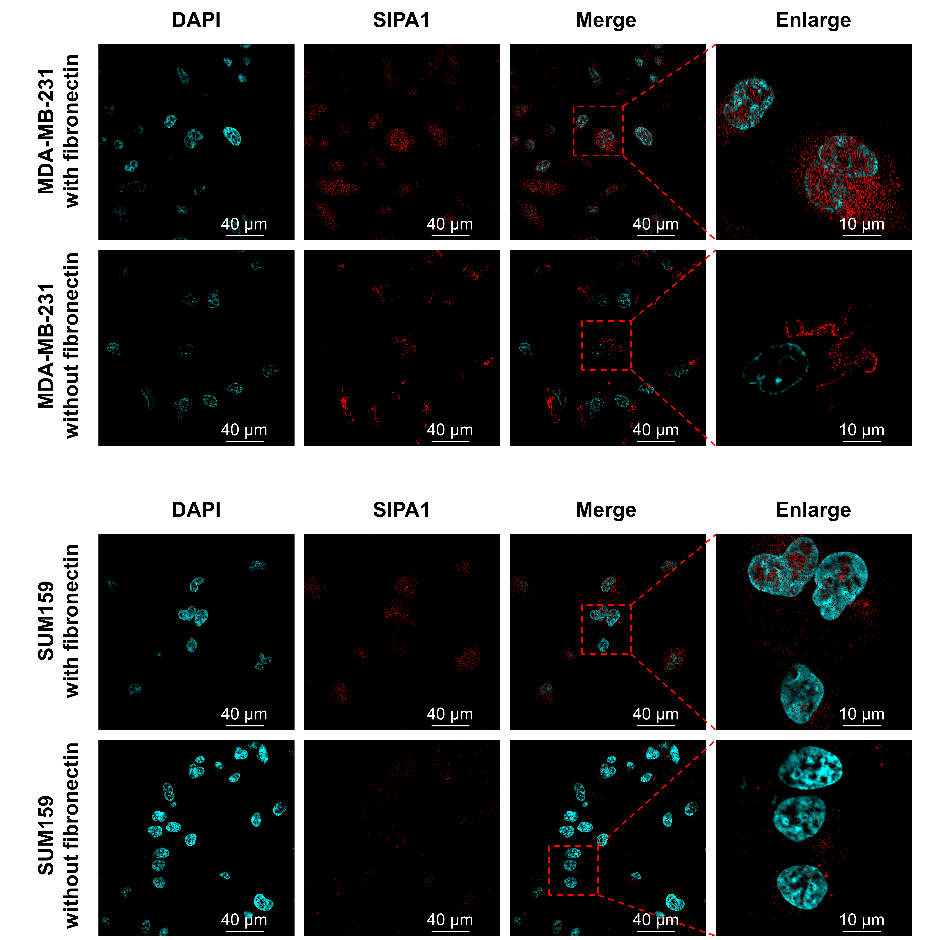


Figure S1. Fibronectin regulates SIPA1 trafficking between the cytoplasm and the nucleus in MDA-MB-231 cells and SUM159 cells.

Fluorescence microscopy imaging of SIPA1 in breast cancer cells MDA-MB-231 and SUM159 which had been starved in FBS-free media and then treated with 5 μg/mL of fibronectin for 12 h. SIPA1 is in red and nuclei in blue (stained with DAPI). Scale bar, 40 μm.


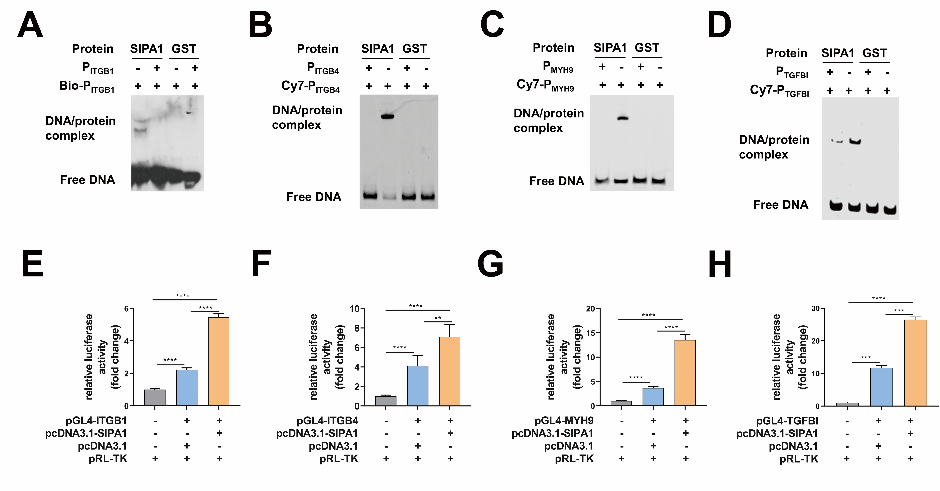


**Figure S2. SIPA1 protein can directly bind to the promoter regions of multiple genes and regulate the transcription activity of these promoters.**

(A-D) DNA binding with SIPA1 was evaluated using EMSA using endogenous SIPA1 proteins and Bio-labeled segment of ITGB1 promoter (A), cy7-labeled segment of ITGB4 promoter (B), cy7-labeled segment of MYH9 promoter (C), and cy7-labeled segment of TGFBI promoter (D).

(E-H) Dual Luciferase assay in HEK293T cells. HEK293T cells were co-transfected with SIPA1 expression plasmids and pGL4.10- ITGB1 (E), pGL4.10-ITGB4 (F), pGL4.10-MYH9 (G), pGL4.10-TGFBI (H) luciferase reporter vector or pGL4.10 (empty). *P*-values were calculated using the unpaired two-tailed Student’s t-test (***p* < 0.01; ****p* < 0.001; *****p* < 0.0001).


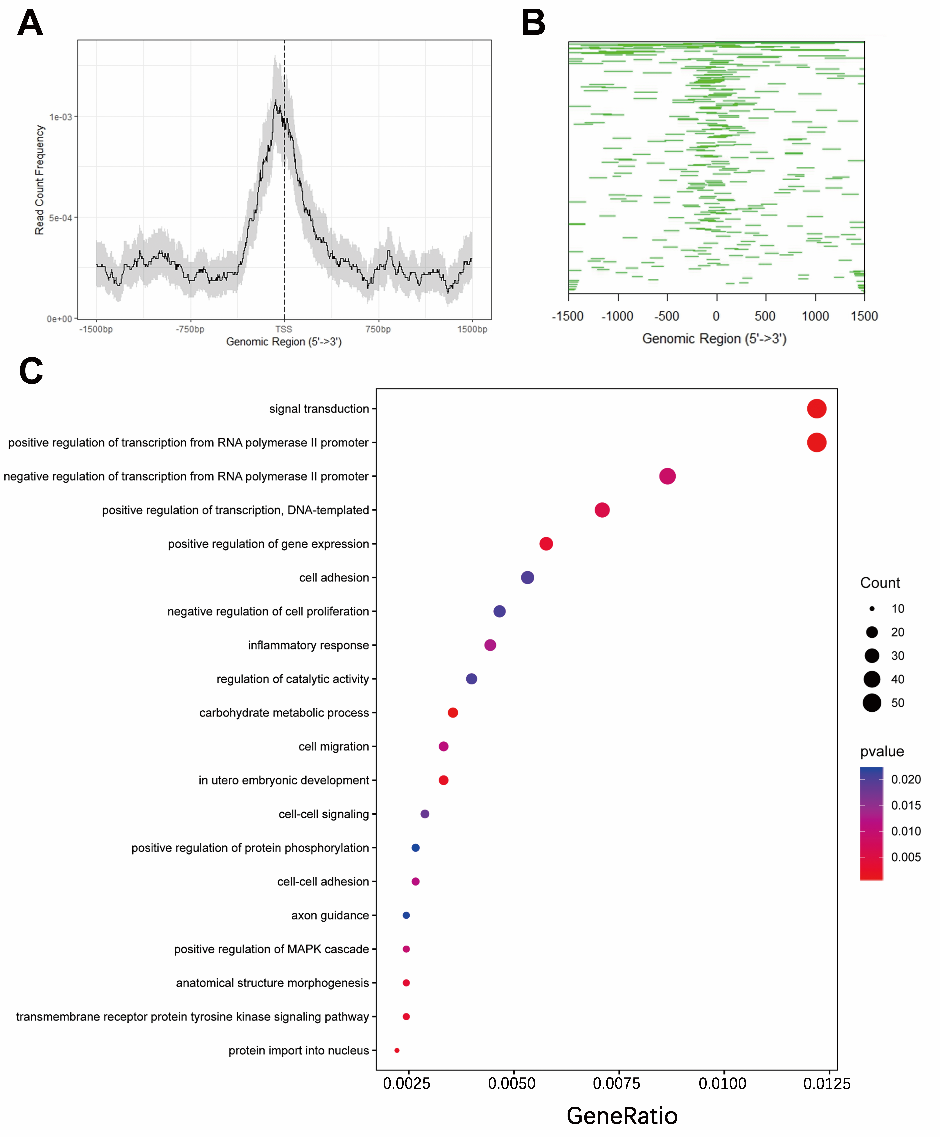


**Figure S3. The SIPA1 protein directly binds to the gene's promoter.**

(A-B) Distance of the SIPA1 protein binding region peaks flanking TSSs throughout the genome. The number of binding region peaks (peak count frequency) corresponding to the ±1,500 bp regions is indicated. (A) Peak plot distribution and（B）Heat map distribution.

(C) Dot plot showing the enrichment analysis for Gene Ontology biological processes among group enrichment analyses of 4,508 genes. The size of dots represents the count of genes, and the spectrum of color indicates the mean *p* values.


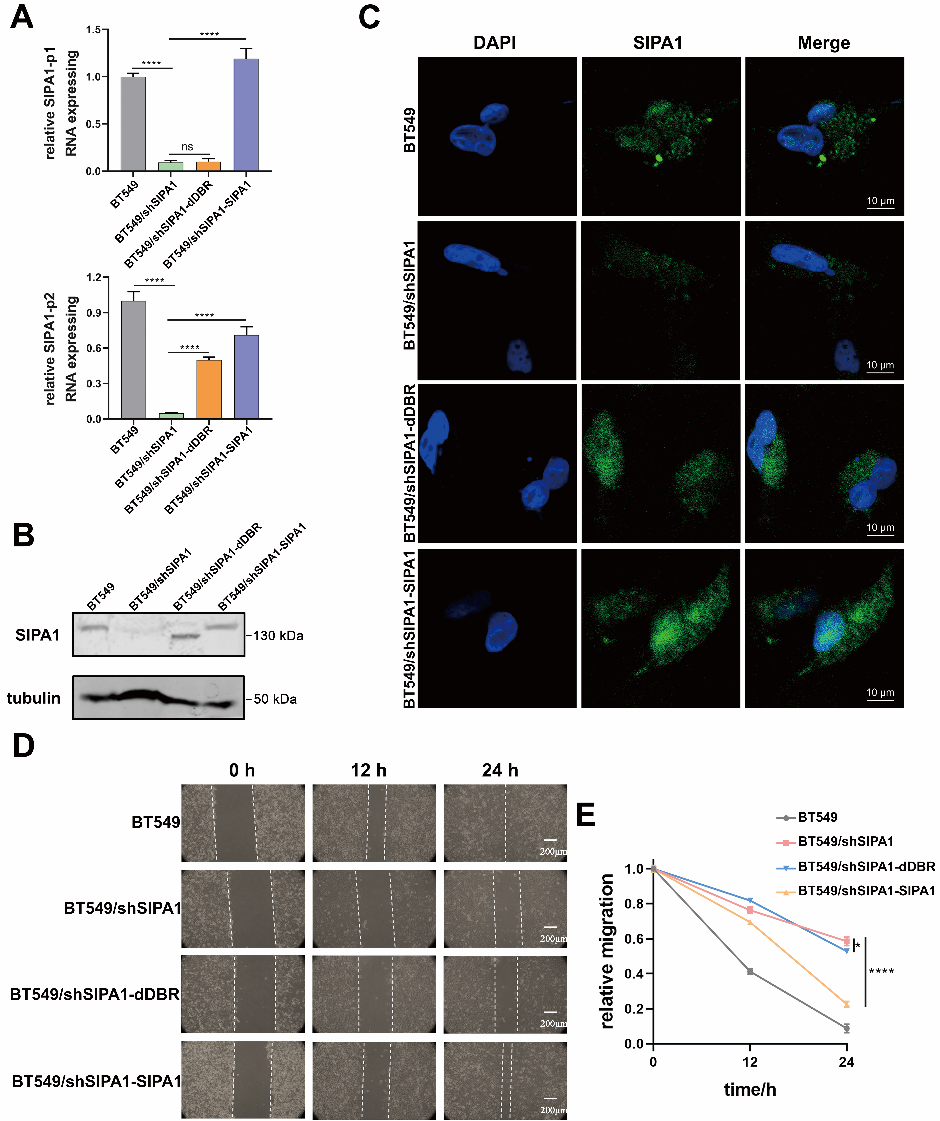


**Figure S4. SIPA1 promote tumor cell migration through DBR.**

(A-B) Expression of SIPA1 and SIPA1-dDBR in breast cancer cells determined by reverse transcription-quantitative polymerase chain reaction (Top: the SIPA1-p1 was in the DBR region; bottom: the SIPA1-p2 was in the PDZ region) (A) and Western blot (B). *P*-values were calculated using the unpaired two-tailed Student’s *t*-test (*****p* < 0.0001).

(C) Fluorescence microscopy imaging of SIPA1 or SIPA1 fragment (dDBR) distribution in different cell lines. SIPA1 protein or dDBR is in green and nuclei in blue (stained with DAPI). Scale bar, 10 μm.

(D-E) Scratch migration assays comparing the closure of wound healing distance in four cell lines, BT549, BT549/shSIPA1, BT549/shSIPA1-SIPA1 and BT549/shSIPA1-dDBR cell lines. Scale bar, 200 μm. (D).and Quantification of the wound healing distance (E). *P*-values were calculated using the unpaired two-tailed Student’s *t*-test (**p* < 0.05; *****p* < 0.0001).


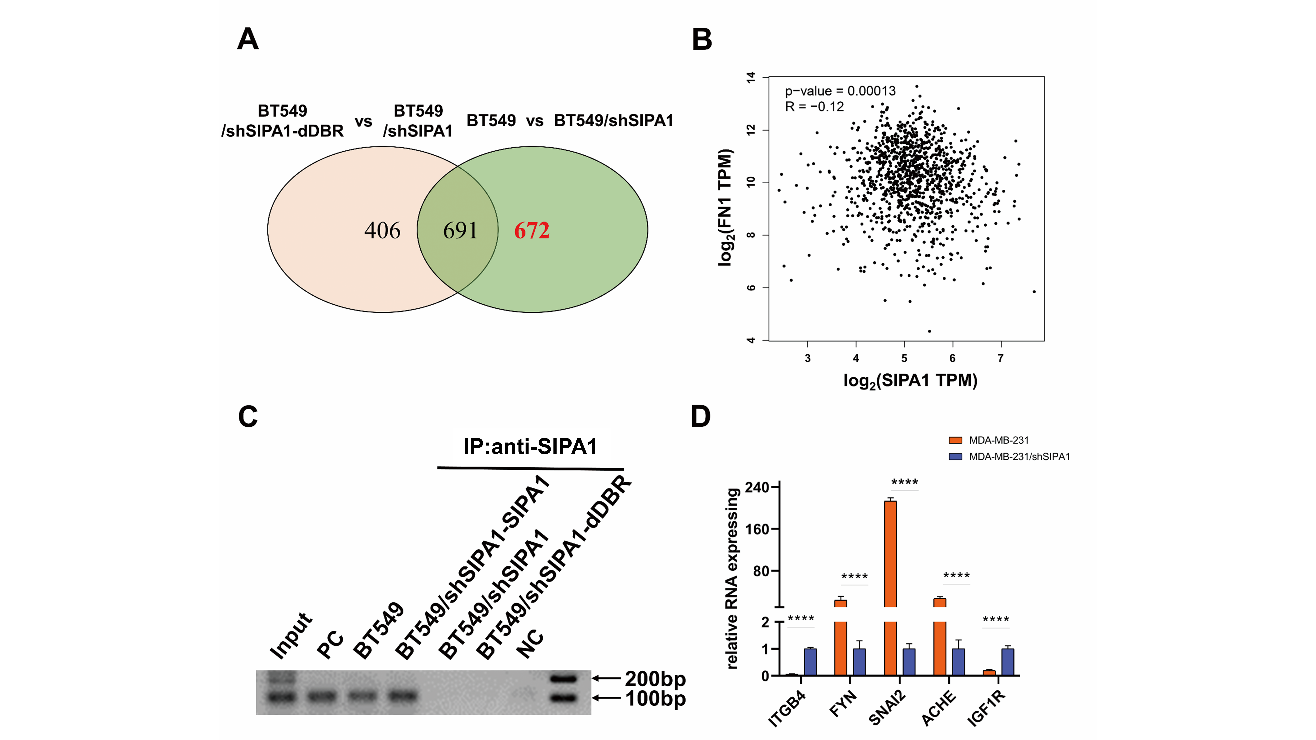


**Figure S5 Correlation between SIPA1 and cell junction organization related protein expression.**

(A) The Venn diagram shows the regulated genes with mRNA expression being significantly changed (fold change > 1.5) by RNA-seq comparing BT549 with BT549/shSIPA1, and BT549/shSIPA1-dDBR, and 672 overlapping candidate genes which were regulated by SIPA1 through the DBR domain.

(B) FN1 expression patterns was analyzed based on TCGA.

(C) SIPA1 binding to the promoter of FN1 gene was assayed by ChIP. Crosslinked nuclear extracts of cells were immunoprecipitated by an anti-SIPA1 antibody. Genomic DNA of BT549 cells and water were used as positive (PC) and negative controls (NC) in the PCR.

(D) mRNA levels of migration-related genes were determined by RT-qPCR . Data were shown as means ± SEM. The experiments were conducted in triplicate. Glyceraldehyde-3-phosphate dehydrogenase (GAPDH) was included as an endogenous control. *P*-values were calculated using the unpaired two-tailed Student’s *t*-test (*****p* < 0.0001).


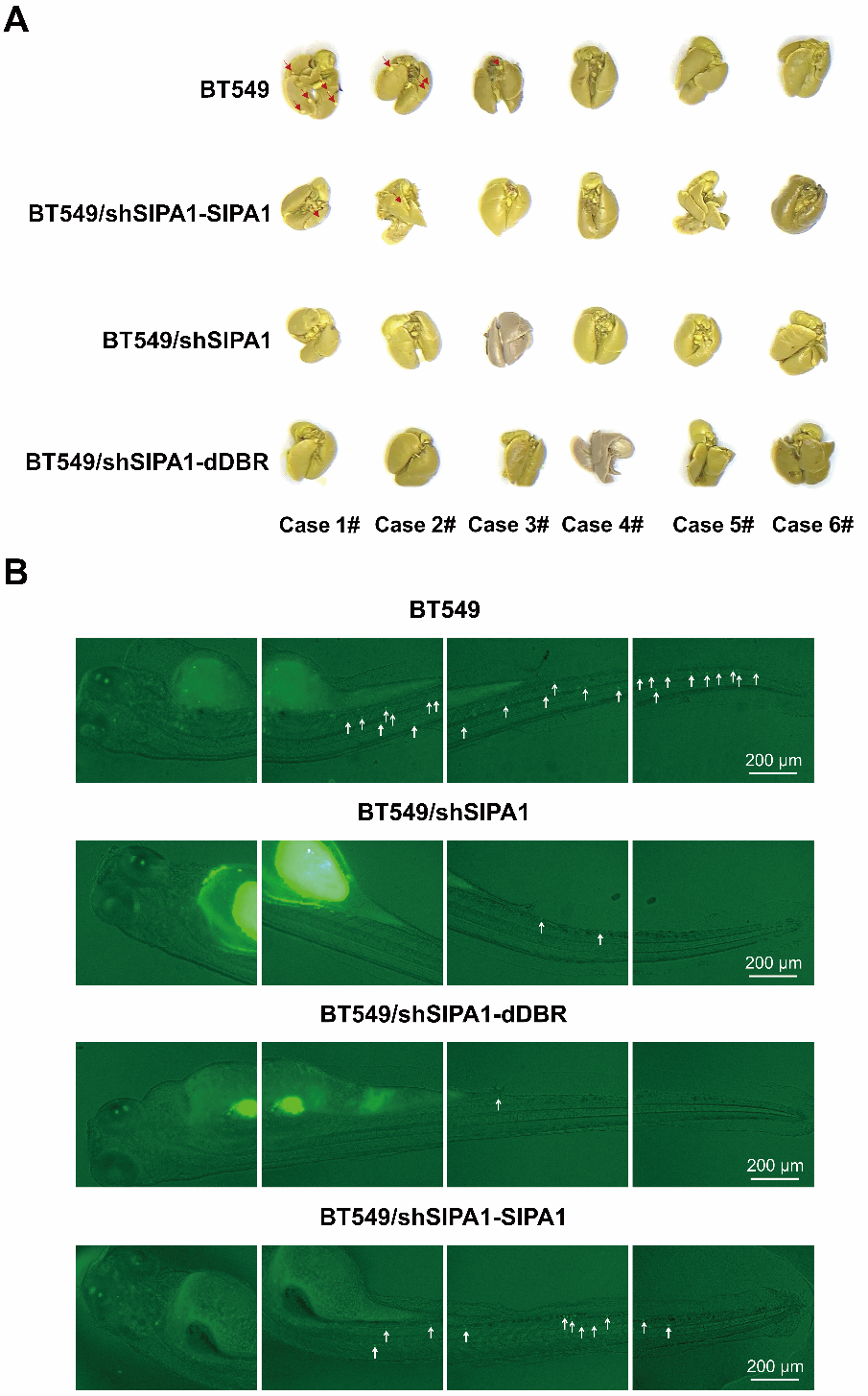


**Figure S6. SIPA1 promotes the breast cancer cell metastasis depending on its DBR structurally.**

(A) Lungs from four groups of human breast cancer cell grafted mice were photographed. Metastatic lesions in the lungs at 26th day after tumor cell injection were shown by Bouin's Fixative solution staining. Red arrows indicated the metastatic tumor nodules.

(B) Fluorescence imaging of the breast cancer cell migration in zebrafish. Dio-labeled breast cancer cells were injected into the yolk sac of zebrafish. Arrowheads indicate disseminated tumor foci (single tumor cells or cell aggregates) in the tail regions. Scale bar, 200 μm.


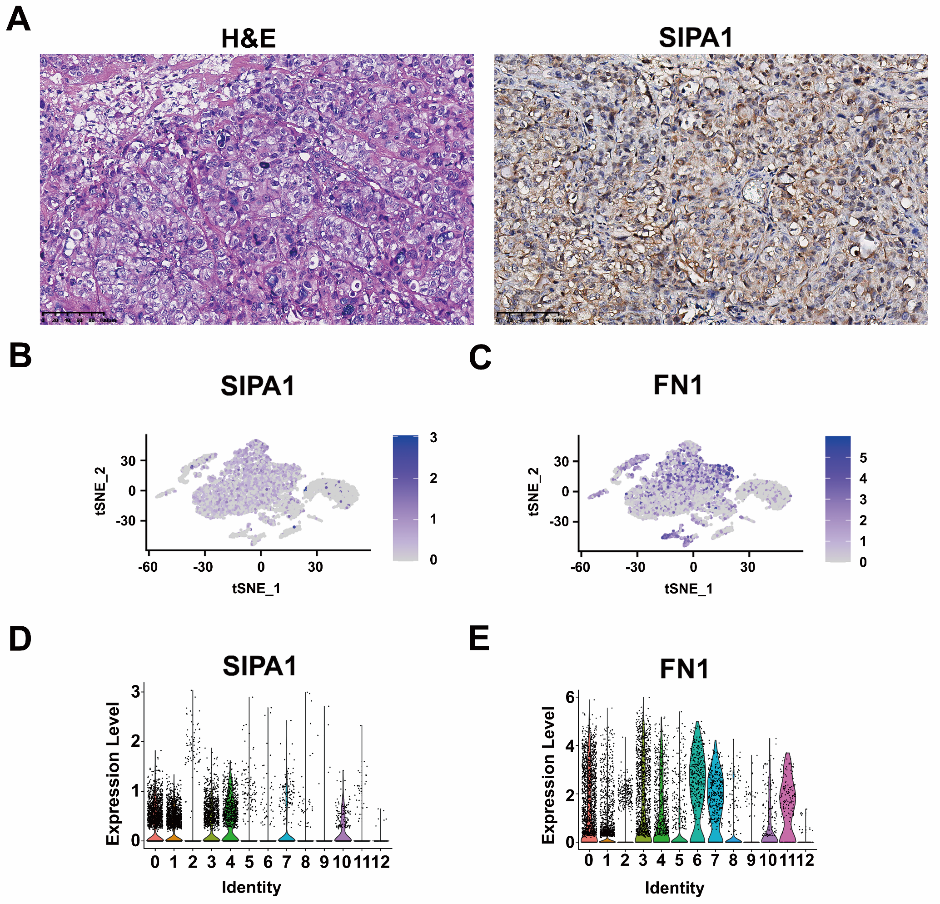


**Figure S7. The expression of *SIPA1* and *FN1* in patient sample based on single cell sequencing data from the breast cancer patient.**

(A) The H&E image (Left) and the image of SIPA1 expression of the breast cancer tissue by immunohistochemistry staining (Right). Scale bar, 100 μm.

(B, C) Feature plots showing the normalized expression levels of SIPA1 and FN1 in the subclusters. The color legend shows the log1p normalized expression levels of the genes.

(D, E) Violin plots showing the normalized expression levels of SIPA1 and FN1 across the 13 clusters.


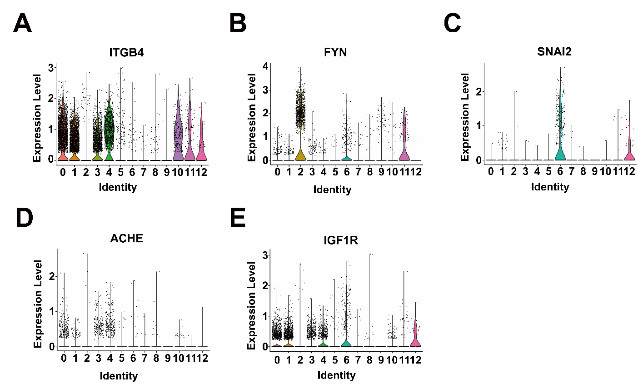


**Figure S8. The expression patterns of *ITGB4*, *FYN*, *SNAI2*, *ACHE*, and *IGF1R* in patient sample based on single cell sequencing data from the breast cancer patient.**

Violin plots showing the normalized expression levels of six genes (*ITGB4*, *FYN*, *SNAI2*, *ACHE*, and *IGF1R*) across the 13 clusters.


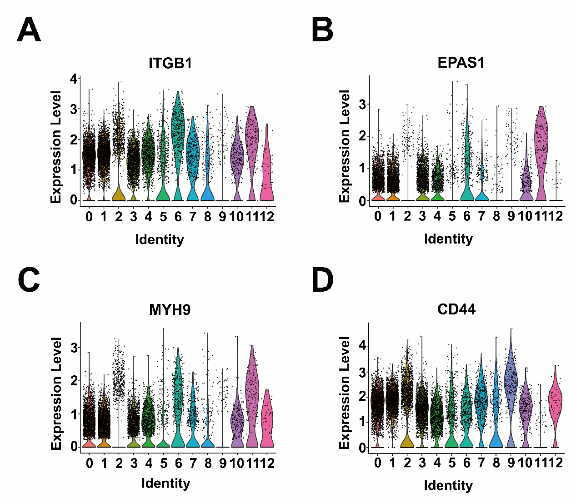


**Figure S9. The expression patterns of *ITGB1, EPAS1, MYH9 and CD44* in patient sample based on single cell sequencing data from the breast cancer patient.**

Violin plots showing the normalized expression levels of four genes (*ITGB1*, *EPAS1*, *MYH9* and *CD44*) previously reported to be upregulated by SIPA1 across the 13 clusters.

**Table S1. SIPA1 bound candidate transport proteins by protein mass spectrometry analysis which related to Figure 1E.**

| **MW** | **Gene** | **Location** | **Function** |
| --- | --- | --- | --- |
| 119516.74 | Tax_Id = 9606 Gene_Symbol = IPO7 Importin-7 | Cytoplasm. Nucleus | Protein transportation |
| 97170.32 | Tax_Id = 9606 Gene_Symbol = KPNB1 Importin subunit beta-1 | Cytoplasm. Nucleus | Protein transportation |
| 104606.72 | Tax_Id = 9606 Gene_Symbol = AP1B1 Isoform A of AP-1 complex subunit beta-1 | Cytoplasm | Protein transportation |
| 91620.07 | Tax_Id = 9606 Gene_Symbol = ITGB1 Isoform Beta-1C of Integrin beta-1 | membrane | Cell adhesion |
| 159864.38 | Tax_Id = 9606 Gene_Symbol = MYH9 Isoform 2 of Myosin-9 | Cytoplasm | Cyoskeleton |
| 113083.66 | Tax_Id = 9606 Gene_Symbol = PARP1 Poly [ADP-ribose] polymerase 1 | Nucleus | DNA repair |
| 116612.38 | Tax_Id = 9606 Gene_Symbol = ITGA3 Isoform Alpha-3A of Integrin alpha-3 | membrane | Cell adhesion |

**Table S2. List of oligo shRNA sequences targeted to SIPA1**

| **Name** | **Sequence (5’-3’)** |
| --- | --- |
| shSIPA1 forward | CCGGTACTACCGCAAATACTTCTATCTCGAGATAGAAGTATTTGCGGTAGTATTTTTG |
| shSIPA1 reverse | AATTCAAAAATACTACCGCAAATACTTCTATCTCGAGATAGAAGTATTTGCGGTAGTA |

**Table S3. Primer list for protein expression**

| **Name** | **Sequence (5’-3’)** |
| --- | --- |
| DBR forward | GGATCTGGTTCCGCGTGGATCCAGCGGCCGGCCCCGCAGGAG |
| DBR reverse | TCACGATGCGGCCGCTCGAGTCAGGCTGTGGTGGCCAGGAGGA |
| PDZ forward | TATCTCGAGATGGATTACAAGGATGACGACG |
| PDZ reverse | TAGGATCCCTCGTCGGGGGGCAGGACGG |
| C1 forward | ATACTCGAGATGGATTACAAGGATGACGACGA |
| C1 reverse | ATGGATCCTCAGGCCAGGTCGGCGGTGG |

**Table S4. Primer list for qRT-PCR**

| **Name** | **Sequence (5’-3’)** |
| --- | --- |
| SIPA1 forward | CAGCTCTCTGTCGGATGAGG |
| SIPA1 reverse | GAGACAAGGTACGTGGCAGA |
| DBR forward | CTTGTCTCTGCGGAACTCCA |
| DBR reverse | GACAGCGACTCCAGAATGGT |
| FN1 forward | ATCTCTGTAATTCAGCTTCAGATGT |
| FN1 reverse | ACATTTCCACCAGCGATGAATGA |
| ITGB4 forward | CGAGGTAGGTCCAGGACGG |
| ITGB4 reverse | GTTTGCCAAGGTCCCAGAGA |
| FYN forward | TTGCGCCATCTGTCAGGAG |
| FYN reverse | CAACCTCGCCTCTACTCTCG |
| SNAI2 forward | CAACGCCTCCAAAAAGCCAA |
| SNAI2 reverse | ACTCACTCGCCCCAAAGATG |
| ACHE forward | CCTGCGCCGGGGAAC |
| ACHE reverse | ATGCCCAGGAAAGCAGAGAC |
| IGF1R forward | GGGGCTCTTGTTTACCAGCA |
| IGF1R reverse | CTCCCGCCTCTCTCGAGTTC |
